# Accuracy of Tests for Diagnosis of Animal Tuberculosis: Moving Away from the Golden Calf (and towards Bayesian Models)

**DOI:** 10.1155/2023/7615716

**Published:** 2023-02-21

**Authors:** Alberto Gomez-Buendia, Pilar Pozo, Catalina Picasso-Risso, Adam Branscum, Andres Perez, Julio Alvarez

**Affiliations:** ^1^VISAVET Health Surveillance Centre, Universidad Complutense de Madrid, Madrid, Spain; ^2^Veterinary Population Medicine Department, University of Minnesota, St Paul, Minnesota, USA; ^3^Facultad de Veterinaria, Universidad de la República, Montevideo, Uruguay; ^4^Biostatistics Program, Oregon State University, Corvallis, Oregon, USA; ^5^Departamento de Sanidad Animal, Facultad de Veterinaria, Universidad Complutense de Madrid, Madrid, Spain

## Abstract

The last decades have seen major efforts to develop new and improved tools to maximize our ability to detect tuberculosis-infected animals and advance towards the objective of disease control and ultimately eradication. Nevertheless, there is still uncertainty regarding test performance due to the wide range of specificity and especially sensitivity estimates published in the scientific literature. Here, we performed a systematic review of the literature on studies that evaluated the performance of tuberculosis diagnostic tests used in animals through Bayesian Latent Class Models (BLCMs), which do not require the application of a (fallible) reference procedure to classify animals as infected with tuberculosis or not. BLCM-based sensitivity and specificity estimates deviated from those obtained using a reference procedure for certain antemortem tests: an overall lower sensitivity of skin tests and serology and a higher sensitivity of interferon-gamma (IFN-*γ*) assays was reported. In the case of postmortem diagnostic tests, sensitivity estimates from BLCMs were similar to estimates from studies based on other methodologies. For specificity, the range of BLCM-based estimates was narrower than those based on a reference test, reaching values close to 100% (but lower in the case of IFN-*γ* assays). In conclusion, Bayesian methods have been increasingly applied for the evaluation of tuberculosis diagnostic tests in animals, yielding results that differ (sometimes substantially) from previously reported test performance in the literature, particularly for in vivo tests and sensitivity estimates. Newly developed models that allow adjustment for relevant factors (e.g., age, breed, region, and herd size) can contribute to the generation of more unbiased estimates of test performance. Nevertheless, although BLCMs for tuberculosis do not require the use of an imperfect reference procedure and are therefore not influenced by its limited performance, they require careful implementation, and transparent systematic reporting should be the norm.

## 1. Introduction

Bovine tuberculosis (bTB) is one of the most important diseases affecting cattle worldwide [1]. Because of its importance, over 636 million euros were invested between 2007 and 2020 in member states of the European Union on national bTB eradication, control, and surveillance programmes [2]. However, the disease is still endemic in several of the contributing member states [3, 4].

One of the main issues associated with the lack of success of eradication programs is the limited performance of bTB diagnostic tests [1, 5]. A recently published meta-analysis showed that estimates of specificity and particularly sensitivity are highly variable; for example, the median sensitivity for the single intradermal comparative cervical tuberculin skin test (SICCT) with standard interpretation reported in this study was 0.50, and was accompanied by a very wide 95% posterior interval (0.26–0.75) [6]. Low sensitivity is problematic since false negative-infected but undetected-animals that are not removed early may perpetuate the disease in a herd. However, false positive animals can be a major issue as well since although reported median specificity estimates for most tests are in general high (above 88%) [6], in scenarios with relatively low prevalence (as in most European regions), the positive predictive value of tests is necessarily low; thus, a limited specificity can lead to the unnecessary culling of noninfected animals and restriction of movements in a herd, undermining the confidence of stakeholders, and increasing the costs associated with the eradication program due to compensations for culled livestock paid by national governments.

The large variability observed in the sensitivity and specificity estimates of bTB diagnostic tests may be due to the wide disparity in study designs, particularly regarding the gold standard reference procedure used to classify an animal as infected or not. Ideally, a gold standard reference must be infallible (sensitivity and specificity equal to 100%). However, this is particularly unrealistic in the case of bTB, since all currently available diagnostic tests have important limitations in their ability to accurately detect infected animals. For instance, bacteriological culture, the most widely used reference procedure in the literature, can have perfect specificity (100%) but typically has low sensitivity, particularly in the early stages of disease [7]. In this context, the use of Bayesian Latent Class Models (BLCMs), which are not based on the comparison of a test result to a reference test, is particularly well suited [8–10]. Still, to date the number of studies that have used BLCMs for estimating the performance of bTB diagnostic tests is much lower than those based on an (imperfect) gold standard.

We conducted a systematic review of the scientific literature to identify studies that used BLCMs to estimate parameters of test accuracy for TB diagnostic techniques in the absence of a gold standard. Our specific aims were: (i) to describe the models and methodologies applied and quality of reporting in this field using published guidelines [11], (ii) to compare estimates of sensitivity and specificity obtained for different diagnostic tests, different species, and with different prior distributions, and (iii) to identify future opportunities to help establish the performance of diagnostic tests currently used in the field in different countries.

## 2. Materials and Methods

### 2.1. Systematic Review and Data Extraction

The literature search review was conducted according to the guidelines in PRISMA [12] and MOOSE [13], and carried out in the search engines PubMed (MEDLINE), Web of Science (WOS), and Scopus on the 14^th^ of March of 2022. The aim was to retrieve studies that assessed the accuracy of diagnostic tests for detection of tuberculosis in livestock or wildlife based on the use of BLCMs in the absence of a gold standard. The search strings used in each database were as follows:

- (Animal OR Animals [MeSH]) AND (Bayes Theorem [MeSH] OR “Bayesian”) AND (“Mycobacterium tuberculosis complex” OR “Mycobacterium bovis” OR “Mycobacterium caprae” OR “bovine tuberculosis” OR “Tuberculosis, Bovine” [MeSH]) (PubMed)
- TS = (Animal AND Bayes AND (“Mycobacterium tuberculosis complex” OR “Mycobacterium bovis” OR “Mycobacterium caprae” OR bovine tuberculosis)) (WOS)
- TITLE-ABS-KEY (animal AND Bayes AND (“Mycobacterium tuberculosis complex” OR “Mycobacterium bovis” OR “Mycobacterium caprae” OR tuberculosis)) (Scopus).

Articles retrieved from each search were exported to a spreadsheet and duplicates were removed. The title and abstract of the remaining articles were screened by two authors (AGB and JA) according to the inclusion and exclusion criteria in Table 1.

References that did not fulfil these criteria according to both reviewers were removed. If there was a disagreement between the reviewers, a third author (PP) made the final decision.

In a second stage, the full text of the remaining references was reviewed by one author (AGB), for the extraction of the following information:

- Year of publication
- Place where the study was conducted (where the population under study was located)
- Host species tested
- Sample size
- Test(s) evaluated, cut-off points used, and kit where applicable
- Statistical model used (number of populations and tests, see below) and assumptions about the conditional independence or dependence between test results
- Statistical software used for Bayesian analysis
- Prior distributions used and source of information for their construction (where applicable)
- Posterior estimates for sensitivity and specificity and (when performed) prevalence
- Use of sensitivity analysis to assess the impact of prior assumptions

The BLCMs used in each study were classified following the methodology proposed by [14] according to the number of tests applied (one, two, etc.), the number of populations under study (one, two, etc.), and whether the test results were assumed to be conditionally independent or dependent. Certain articles included the application of more than one BLCM (or the independent analysis of more than one population/test), in which case they were subdivided into several “trials” (i.e., independent analyses leading to independent results for a given population/test).

**Figure 1:**
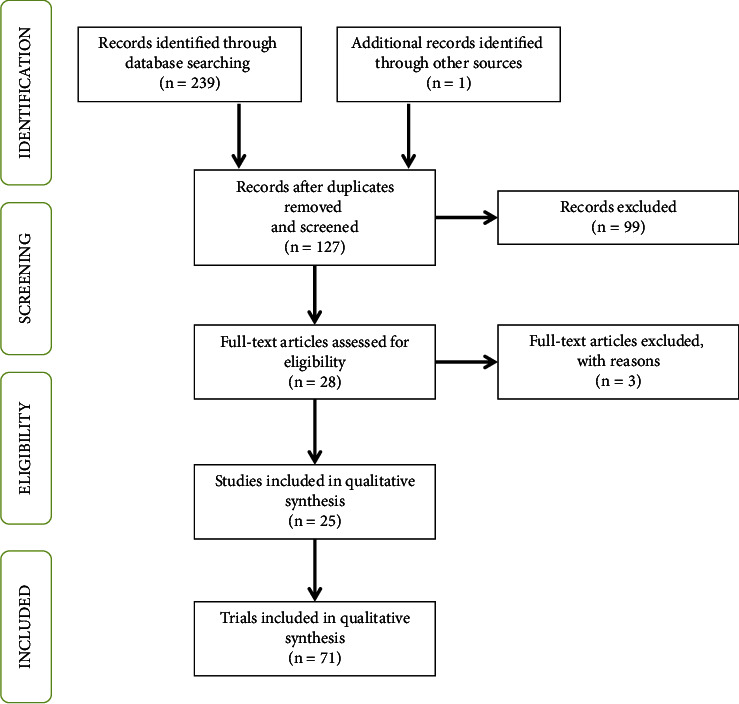
Flow of information followed during the systematic review process.

All relevant references cited in the reviewed articles were also screened following the same process. The flow of information for the different phases of the systematic review is shown in Figure 1.

In order to compare the estimates of different TB tests, an average posterior median (APM) and average 95% posterior probability interval (APPI), considering all median and lower and upper bounds for the 95% PPIs reported for each test, were calculated. To evaluate the relationship between prior and posterior distributions, the Spearman correlation coefficient between their 95% probability interval widths (PIWs) was calculated. For this, 95% prior PIWs were obtained by generating 100.000 random numbers from their beta distributions. The PIWs of the posterior distributions were calculated using the “epi.betabuster” function from the R package “epiR” [15] using the median and 5% percentile reported in the studies. Further, the degree of similarity between prior and posterior distributions was measured by estimating the percentage of overlap between the distributions using an in-house overlap function in R [16] (Supplementary Material 1). In addition, the Spearman correlation coefficient between the 95% posterior PIW and the sample size was calculated. All statistical analyses were performed in R [16].

### 2.2. STARD-BLCM Guidelines

Articles were assessed against the Standards for the Reporting of Diagnostic accuracy studies that use Bayesian Latent Class Models (STARD-BLCM [11], https://www.equator-network.org/reporting-guidelines/stard-blcm/). The STARD-BCLM guidelines consist of an extension of the original STARD-checklist [17] developed to facilitate complete and transparent reporting of diagnostic accuracy studies based on BLCMs in the absence of a gold standard. Three authors (AGB, CPR, and PP) evaluated in parallel each of the full texts selected in the literature review to determine whether they met each of the standards described in the checklist. When there was a disagreement, the options were discussed, and a consensus decision was reached.

## 3. Results

A total of 239 references were identified using the search engine programs (PubMed: *n* = 84; WOS: *n* = 74; Scopus: *n* = 81). Additionally, one European Food Safety Authority (EFSA) scientific opinion was included in the review, leaving 127 records after duplicates were removed.

After the first screening, 99 records were excluded because they discussed topics or were focused on populations not related with the objective of this review (animal movement and within and between-herds transmission models [*n* = 21, 21.2%], *Mycobacterium tuberculosis* complex genomics [*n* = 18, 18.2%], diagnostic test evaluation for other mycobacterial species [*n* = 13, 13.1%], Bayesian models for drug development against TB [*n* = 11, 11.1%], diagnostic tests for TB in humans [*n* = 9, 9.1%], spatial-temporal variations in animal TB [*n* = 9, 9.1%], new statistical methods for the evaluation of diagnostic tests within a Bayesian context [*n* = 5, 5.1%], and 13 other reasons affecting one article each [*n* = 13, 13.1%]).

Out of the 28 articles with the full text reviewed, three were discarded: two described the use of Bayesian models for a purpose other than the assessment of sensitivity and specificity in the absence of a gold standard [18, 19], and the third evaluated a novel BLCM by using TB as an example and not as the subject of study [20]. Consequently, 25 articles (19.7% out of the references initially found) were included in the systematic review.

The characteristics of the studies included in the systematic review are presented in Table 2. All 25 studies were published after January 2009 and originated from Europe (*n* = 11, 44%), North and South America (*n* = 6, 24.0%), Africa (*n* = 4, 16.0%), or Asia (*n* = 4, 16.0%). Most studies (*n* = 19, 76.0%) were focused on cattle, while the remaining were based on the analysis of wildlife (wild boar, badger, bison, meerkat, and elk) and swine.

Approximately two-thirds of the studies considered either only antemortem (*n* = 12, 48.0%) or postmortem tests (*n* = 5, 20.0%), whereas eight studies assessed the performance of both antemortem and postmortem tests (32.0%).

The antemortem tests evaluated in the studies were typically skin and serology-based tests (assessed in 13 articles each, 65.0% of the 20 studies considering antemortem tests) and IFN-*γ* assays (*n* = 11, 55.0%). Postmortem tests included direct polymerase chain reaction (PCR) tests (*n* = 10, 76.9% of the 13 studies considering postmortem tests), bacteriological culture (*n* = 9, 69.2%), meat inspection (*n* = 5, 38.5%), histopathological examination (*n* = 2, 15.4%), or a combination of culture and meat inspection (*n* = 1, 7.7%).

Bayesian modelling was typically implemented using WinBUGS (13/25, 52.0%), with the remaining studies using OpenBUGS (*n* = 7, 28.0%) or JAGS (*n* = 5, 20.0%). Priors for the sensitivity and specificity of each test under evaluation were generally based on previous literature (13, 52.0% of all the studies) or a combination of previously published estimates with expert opinion (7, 28.0%), while weakly informative priors were used for all tests evaluated in the remaining five (20.0%) studies. In 12 articles (48.0%), diffuse priors (uniform (0, 1) distributions) were used for the sensitivity and/or specificity of at least one test. Eighteen articles (72.0%) included a sensitivity analysis based on the replacement of informative priors by diffuse priors to assess the impact of prior selection as an additional analytic step.

Multiple studies (15, 60.0%) included more than one analysis (e.g., different models applied in different populations/tests), and therefore the 25 articles were divided into 71 trials. Among them, over two-thirds (49, 69.0%) considered conditional dependence between test results. Out of these, more than two-thirds (32/49, 65.3%) assessed the use of a skin test and an IFN-*γ* assay in parallel and based the assumption of conditional dependence on the consideration that both tests are designed to detect a cell-mediated immune response.

### 3.1. Antemortem Tests

**Figure 2:**
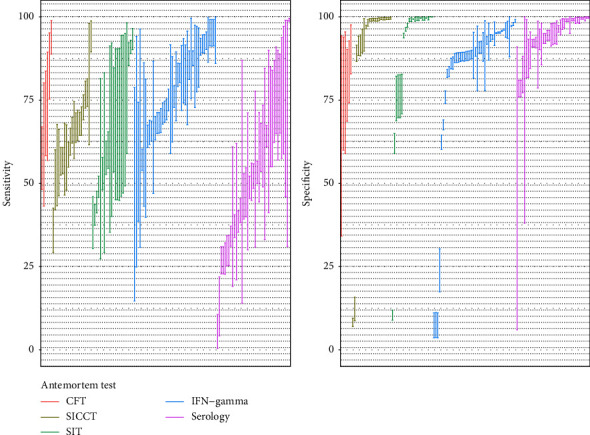
95% posterior probability intervals for the sensitivity and specificity estimates obtained for antemortem tests in the studies retrieved in this study.

The 264 Bayesian estimates of the performance of antemortem TB diagnostic tests extracted from the studies selected in the literature review are summarized in Table 3. One hundred and sixty (71.4%) of these estimates were obtained using BLCMs with informative priors. The posterior estimates for the sensitivity of skin purified protein derivative (PPD)-based tests had a wide range of values, varying between 40.0% and 93.0% (Figure 2). The highest APM sensitivity estimate (72.9%) among the skin-based tests was obtained for the caudal fold test (CFT). When only studies in cattle were considered, the APM for the sensitivity of CFT was similar (73.3%; 95% APPI: 58.3–89.4%). For specificity, overall the cervical skin tests had an APM of 99.2% (95% APPI: 98.7–99.5%), in contrast to CFT, which had the lowest APM (78.7%, 95% APPI: 64.3–94.1%).

Posterior estimates of the sensitivity and specificity of IFN-*γ* tests were highly variable depending on the kit used. BOVIGAM™ TB Kit (BOVIGAM) yielded a much higher estimated sensitivity compared to the ID Screen® Ruminant IFN-*γ* (IDVet, evaluated in just one study) and, in contrast, had a lower estimated specificity. Most of the APM sensitivity estimates for IFN-*γ* tests were higher than for skin tests, except for the only study assessing the performance of the IDVet test [21]. However, the APM estimates of specificity for IFN-*γ* tests were lower than those for skin tests (Figure 2).

In the case of serology-based tests, a wide range of estimated sensitivities and specificities was observed (Figure 2), potentially due to the large variety of species in which these tests were applied. In general, lower sensitivity estimates were reported than for the other antemortem tests, and higher estimated specificity was reported compared to those obtain for IFN-*γ*, but lower than for skin tests. These and additional numerical summaries are presented in Table 3.

Overall, posterior probability intervals were narrower for specificities than for sensitivities for all the assessed tests (Figure 2).

### 3.2. Postmortem Tests

**Figure 3:**
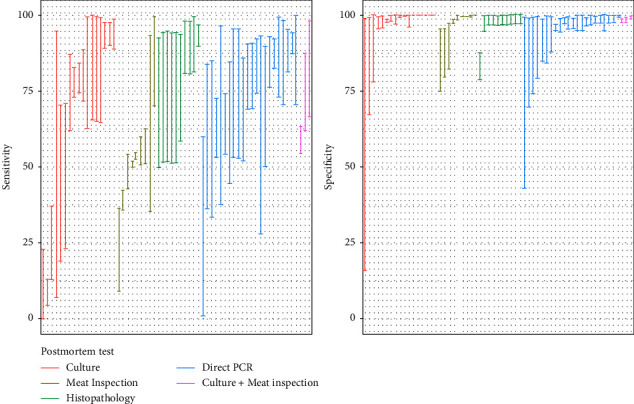
95% posterior probability intervals for the sensitivity and specificity estimates obtained for postmortem tests in the studies retrieved in this study.

Thirteen articles evaluated the performance of TB postmortem tests using BLCMs, of which 120 estimates were extracted. Of these, only 30.0% (36/120) were determined with Bayesian models that used informative prior distributions. The posterior probability intervals for the sensitivity of the postmortem tests were wide, while the posterior medians of specificity were 100% in most of the studies (Figure 3 and Table 4). Direct PCR had the highest APM sensitivity among all the postmortem tests (80.6%; 95% APPI: 54.4–92.3%), while meat inspection had the lowest (53.7%; 95% APPI: 49.9–54.8%). A higher APM for sensitivity was reported for cattle populations in the case of bacteriological culture, with an estimated sensitivity of 88.9% (95% APPI: 65.5–97.5%) compared to an overall estimate of 79.2% (95 APPI: 64.8–88.7%).

### 3.3. Prior and Posterior Distributions

**Figure 4:**
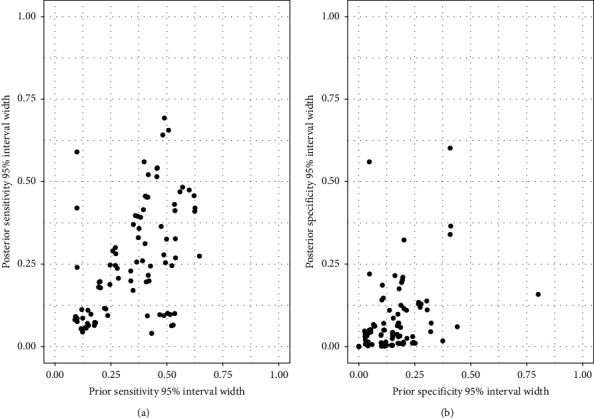
Scatterplots of 95% prior and posterior interval widths for sensitivity (a) and specificity (b) of diagnostic tests for tuberculosis in studies that used informative prior distributions.

For studies that used informative prior distributions for sensitivity and specificity parameters, the correlation between 95% prior and posterior probability interval widths was 0.53 for sensitivity and 0.41 for specificity (Figure 4). In general, prior probability intervals were wider for both sensitivity (median PIW = 0.37; interquartile range (IQR): 0.18–0.47) and specificity (median = 0.15; IQR: 0.06–0.20) than posterior probability intervals (median for sensitivity = 0.24, IQR: 0.10–0.40; median for specificity = 0.04; IQR: 0.01–0.10).

**Figure 5:**
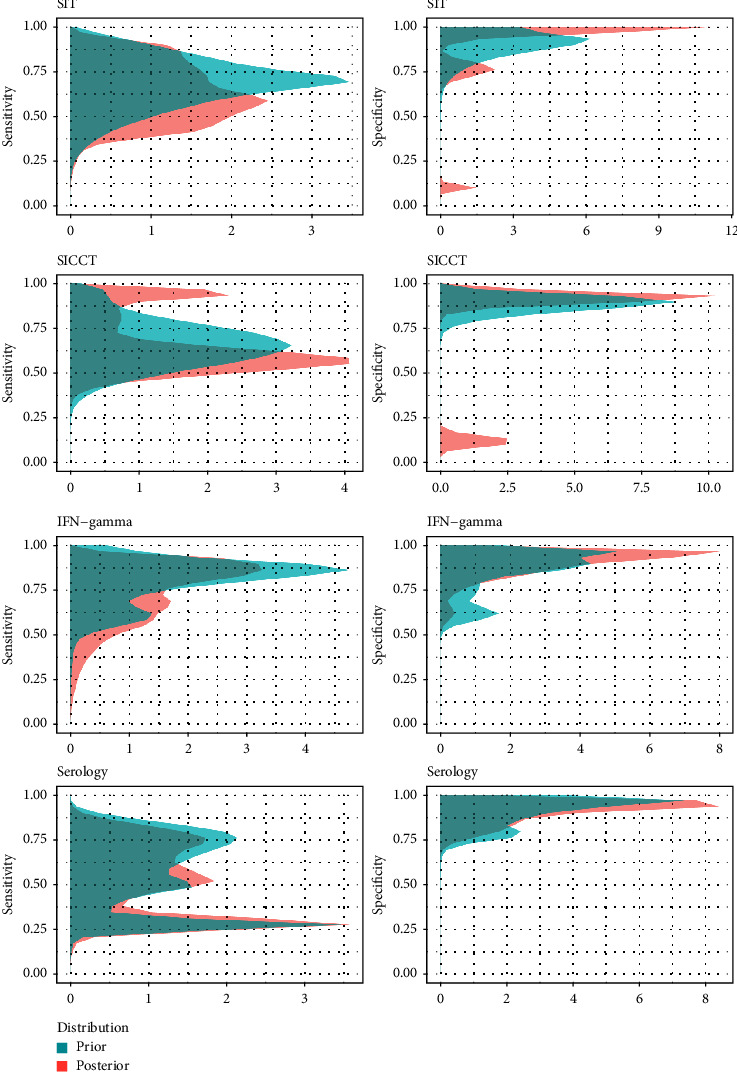
Combined prior and posterior distributions of sensitivity and specificity estimates of antemortem diagnostic tests for tuberculosis obtained using informative priors (left: sensitivities; right: specificities). Each trial was set to have the same weight. This information is shown by trial in Supplementary Material 3.

**Figure 6:**
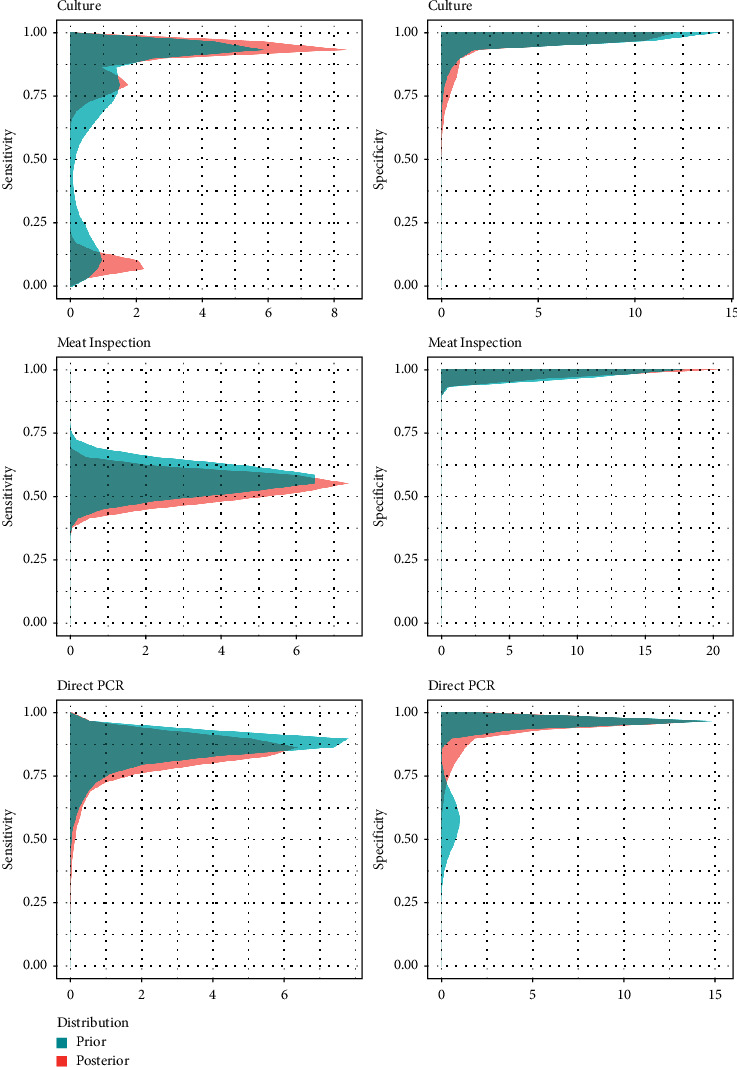
Combined prior and posterior distributions of sensitivity and specificity estimates of postmortem diagnostic tests for tuberculosis obtained using informative priors (left: sensitivities; right: specificities). Each trial was set to have the same weight. This information is shown by trial in Supplementary Material 3.

The overlap between prior and posterior distributions was higher for sensitivity (median overlap = 61.0%; 95% range: 55.3–66.7%) than for specificity (29.9%; 95% range: 23.6–36.2%), indicating that posterior distributions shifted away from the corresponding informative prior distributions as a result of information supplied by the data, particularly for the specificity (Figures 5 and 6). Lastly, there were moderate negative correlations between sample size and PIW for sensitivity (*r* = −0.27) and specificity (*r* = −0.64) (Supplementary Material 2).

### 3.4. STARD-BLCM Checklist Review

Fourteen (56.0%) articles were published after the release of the STARD-BLCM guidelines in 2017. Three of these articles submitted the checklist as supplementary information, while four others stated that the guidelines were followed.

**Figure 7:**
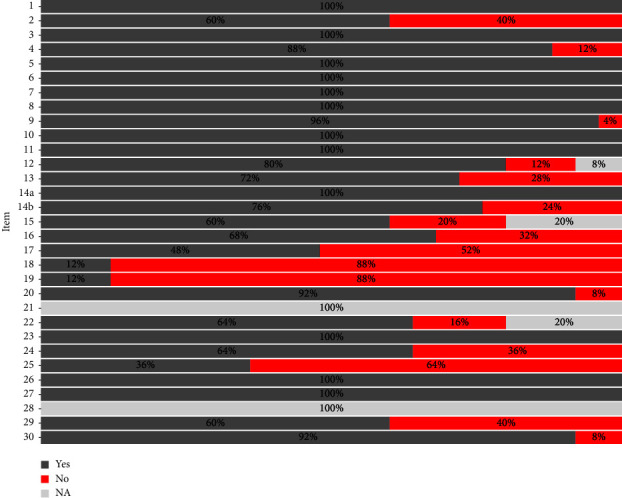
Results of the STARD-BLCM checklist review by the authors. NA refers to articles where the item was not applicable.

Overall, 570 (81.4%) of the 700 possible evaluations performed on the articles selected in the systematic literature review (28 items applicable assessed in 25 articles) were considered fulfilled. The item that was most often not addressed satisfactorily was number 18 (“Intended sample size and how it was determined”), with only three studies reporting it. Also, items 19 (“Flow of participants, using a diagram”), 25 (“Report any adverse events from performing the tests under evaluation”), and 17 (“Any analyses of variability in diagnostic accuracy”) were seldom reported (12.0%, 36.0%, and 52.0%, respectively) compared with the other items (Figure 7). As stated in the STARD-BLCM checklist, item number 21 (“Distribution of the targeted conditions”) was not applicable in the evaluated studies, as the target condition to be detected is unknown, as well as item number 28 (“Registration number and name of registry”) as it applies to clinical trials, which is not the case for the studies assessed here.

## 4. Discussion

The limitations of currently available TB diagnostic tests are among the main factors hampering disease control [22, 23]. This problem is exacerbated by the lack of consensus on the accuracy of TB diagnostic tests, with widely different estimates being reported, likely due to differences in study design and study populations, protocols followed, and reference test used (or not) in the analysis. Given the limitations of all available reference tests for TB, here our purpose was to review the literature on the performance of TB diagnostic tests for studies that used Bayesian analysis in the absence of a reference procedure.

Even though there is still a limited number of studies using Bayesian latent class methodology compared to traditional gold-standard-based approaches (6 out of 113 papers found in a systematic review published in 2018 [6]), Bayesian methods have become more widespread in the last decade, with 14 of the 25 studies retrieved in this literature review being published in the last four years. As expected, studies were performed mostly on cattle (19/25), although several well-known wildlife reservoirs such as wild boar, deer, and badger were also considered (one article each). Interestingly, goats were not the subject of any study in our review, even though they constitute an important reservoir in several regions of the world [24–27]. Also as expected, diagnostic tests based on a cellular immune response were the most commonly assessed techniques, although the increasing usefulness of BLCMs for test evaluation is also demonstrated by its application in the new generation of serology-based tests recently developed for TB (13/25), which were the subject of most studies involving wildlife (5/6) [28, 29] in our review.

Although all studies included in our literature review used BLCMs for data analysis, several differences regarding the priors and specific models considered were found. Regarding prior distributions, almost half of the articles used diffuse priors for at least one of the tests considered, and five out of the 25 studies used only diffuse priors. While diffuse priors may be a reasonable option for the evaluation of newly developed tests [30], four of the five studies that used only diffuse priors evaluated well-established diagnostic techniques (skin tests or IFN-*γ* assays), and therefore published information or expert opinion was likely available [6, 29, 31]. Still, the impact of (informative) priors should always be assessed through a sensitivity analysis [11, 32], which was not done in seven out of the 25 studies.

The development of statistical software that allows fitting BLCMs has been crucial in increasing the application of these approaches. All the studies here were based on BLCMs with codes that are publicly available (e.g., https://cadms.vetmed.ucdavis.edu/diagnostic/software [14]), and were implemented using open-source Gibbs samplers (WinBUGS, OpenBUGS, and JAGS). Interestingly, the use of Stan [33, 34], another open-source statistical modelling software that allows fitting complex Bayesian models [35–37], has not been explored to date in our context.

Results for all antemortem and postmortem techniques were consistent to some extent, suggesting that in general most techniques (with the possible exception of CFT and the IFN-*γ* assay) had high specificity (>90% in 141/192 estimates) while sensitivity estimates were lower and more highly variable. Average posterior median estimates of the sensitivity of skin tests were at or below 70% except for CFT (73%). These estimates are lower than those obtained from the literature based on the use of a reference test, particularly for the SIT test, for which the median estimated sensitivity reported in a recent meta-analysis was 81% or 94% depending on the analysis [6], compared to the 64–70% range from BLCM-derived estimates described here. In the case of SICCT, Bayesian estimates of sensitivity were more consistent across studies than published frequentist estimates, with the former ranging between 52.5 and 66.6% (APPI for standard interpretation) or 66.8 and 75.6% (APPI for severe interpretation), whereas frequentist estimates ranged between 50 and 100% [5, 6, 31]. In contrast, Bayesian estimates for the specificity of the skin tests reviewed in this study agreed with previously reported values close to 100% [31, 38] with the exception of CFT. In this case, the frequentist and Bayesian estimates are different for both sensitivity and specificity, with frequentist estimates being higher (85.7% mean vs. 72.9% APM for sensitivity, and 92.8% mean vs. 78.7% APM for specificity) [39]. Counterintuitively, according to the APM, the SIT test using a severe interpretation was the most specific skin test, what could be due to the specific animal populations being tested, but in any case, APPIs for the specificity of most skin tests were largely similar (Table 3).

Interestingly, Bayesian estimates of sensitivity reported for IFN-*γ* tests were in general higher and more consistent (ranging from 71 to 90% [95% APPI]) than those obtained through comparison with a gold standard (with a 95% posterior interval of 49–82%) [6]. This could be in part due to the more recent nature of Bayesian studies that thus would have (i) been based on more optimized IFN-*γ* tests and (ii) evaluated using similar cut-off points (as opposed to some of the references based on a gold standard, some of which were published before the 2000s and were often based on widely different interpretation criteria). In contrast, Bayesian estimates of specificity were lower than those obtained using a gold standard. Some of these were based on testing officially tuberculosis free (OTF) populations, what would indicate that these OTF herds were systematically different in some unknown characteristics (or exposure to certain antigens) from the infected herds where IFN-*γ* test have been typically applied, thus limiting the external validity of these studies. Nevertheless, to date, few articles have studied the use of IFN-*γ* in OTF herds, and these were based on the use of different commercial kits, cut-offs, and population characteristics (e.g., production type and age) [40–43]. Other estimates based on the use of a gold standard (typically a postmortem test such as bacteriological culture) with limited sensitivity may have led to the misclassification of some truly infected animals and therefore should be interpreted with care since this would lead to underestimating the specificity of the test.

A wide range of serology-based test sensitivities were estimated through BLCMs (Figure 2), which could be attributable to the large number of species to which these tests were applied. BLCM-derived sensitivity estimates were lower than those obtained in previous studies, which reported sensitivity estimates above 63% [28, 44–47] when using bacteriological culture and the presence of visible lesions as the gold standard, or up to 75% when compared with IFN-*γ* [45, 48]. In contrast to the articles included here, these studies were performed in animal populations with a suspected high prevalence (i.e., high proportions of skin test or IFN-*γ* reactors, herds subjected to depopulation due to a TB outbreak) or subjected to experimental infections [46, 48]. Serology tests are known to perform very well in these high prevalence settings where the infection is typically at an advanced stage [49, 50], which could lead to an overestimation of their sensitivity for lower prevalence populations. In fact, sensitivity estimates below 35% have been reported in low-prevalence settings [28, 51], demonstrating how sensitivity varies depending on the prevalence of the population tested. Furthermore, sensitivity is known to increase due to the anamnestic effect of a skin test prior to the sampling, which was the case for some studies in our review [28, 46, 48], and this could also partially explain the difference between the estimates presented here and in previous literature. [28, 51] In contrast, specificity estimates obtained using BLCMs (ranging between 90–100%) were aligned with previous estimates obtained from TB-free cattle herds [5, 6, 28, 46, 48] and wildlife populations [52–54].

Regarding postmortem tests, meat inspection (i.e., abattoir surveillance) had the lowest sensitivity (53.7%; 95% APPI: 49.9–54.8%). BLCM estimates were consistent with other estimates [55, 56], with values at or below 60%, although other studies have reported even lower (<40%) estimates [57–60]. Differences between estimates from these studies may be attributable to multiple factors, such as the quantity and training of inspectors and the amount of time spent on each animal inspection [60–62], rather than to the methodology used to assess test performance, given that this diagnostic test is particularly difficult to standardize under field conditions. Regardless, passive surveillance has an important role as a monitoring tool, particularly when disease is absent or at very low levels, and therefore the continued assessment of its performance is highly relevant [63, 64]. Bacteriological culture has traditionally been used for the confirmation of TB and as a reference for the evaluation of other diagnostic techniques. Nowadays, direct PCR has been introduced in many laboratories as an alternative to culture for TB confirmation. Interestingly, both tests showed similar APM in terms of sensitivity, although specificity was slightly lower for direct PCR (but still close to 100%). Frequentist-based sensitivity estimates described in the literature are variable, ranging from 30 to 100% for culture [65–68] and 63 to 100% for direct PCR [69–72]. This heterogeneity between estimates from different studies was also observed in BLCM-based estimates (Figure 3), with median posterior sensitivity values for culture and direct PCR in tissues varying between 8 and 97% for culture and 61 and 91% for direct PCR depending on the study. These wide ranges could be due to the influence of several factors such as the presence of compatible visible lesions and differences in sample collection and preservation, as well as in the protocol followed [65, 66, 71, 73]. In any case, the similar diagnostic performance of culture and direct PCR found in our analyses, coupled with the significantly lower turnaround time for obtaining the results of the latter (days versus weeks), suggests direct PCR can be a useful alternative for postmortem confirmation of TB in the frame of eradication programs as evidenced by its inclusion in Commission Delegated Regulation (EU) 2020/689 (Article 9) as an official diagnostic test.

Even though results obtained for each of the diagnostic tests evaluated were somewhat consistent, indicating a higher sensitivity of IFN-*γ* assays compared with skin and serology tests while the specificity would be higher for skin tests, there was considerable variation between studies, with e.g., median sensitivity estimates for the IFN-*γ* assays (BOVIGAM) ranging from 55.7% to 95.8% (Figure 2). This could be due to the effect of local factors such as the presence of nontuberculous mycobacteria, herd size or the production type, and age of the animals, which also can influence estimates obtained through the comparison with a reference procedure [74–76]. However, the limited number of studies based on BLCMs conducted so far makes the identification of these local factors and the characterization of their impact challenging; nevertheless, they should be considered when interpreting the results.

Transparency in reporting is key for the evaluation of a study [77–80]. In the case of TB test accuracy BLCM studies, only one-fifth of the articles published after the STARD-BLCM guidelines were released (3/14 studies) provided the checklist as supplementary material, with other four indicating the guidelines were followed. Among the more problematic items in terms of compliance, the most relevant one was the lack of justification for sample sizes (only three out of 25 articles) and the assessment of variability in test performance in subpopulations, which could be important in the case of TB given the known effect of certain host characteristics (e.g., age and breed) [63, 81, 82] for which there is often available information but that was seldom incorporated in the analysis. Recently developed models that allow for the inclusion of covariates in the context of BLCMs could be a suitable analysis option [82–84]. Other items that were underreported, such as the flow diagram of participants or the occurrence of adverse events associated with test administration, are probably less significant in the assessment of TB diagnostic tests, which could explain why they were typically not reported.

## 5. Conclusions

Results from studies that used BLCMs to assess the performance of TB antemortem diagnostic tests deviated consistently from those obtained from analyses that used (imperfect) reference procedures, particularly with regards to their sensitivity: Bayesian posterior estimates of sensitivity were overall lower for skin tests and serology and higher for IFN-*γ* assays. In contrast, estimates based on BLCMs and the use of a reference test mostly agreed on the performance of postmortem TB diagnostic tests. Given the limitations of all available reference procedures for TB, BLCM-based estimates may more accurately reflect the performance of tests in the field, though the high variability observed between studies suggests test performance may be affected by multiple factors not related with the use of an imperfect reference procedure. Newly developed models that allow for the inclusion of some of these (often spatially structured) factors may help to produce accurate estimates of test performance in the future, thereby informing and optimizing control and eradication programs based on test-and-cull strategies.

## Figures and Tables

**Table 1:** Inclusion and exclusion criteria.

Inclusion criteria	Exclusion criteria
Articles written in English or Spanish	Other languages
At least one diagnostic test for detection of tuberculosis infection (caused by a member of the *Mycobacterium tuberculosis* complex) is evaluated	Considered tests aimed at the detection of infection due to nontuberculous (myco) bacteria
Diagnostic tests are applied in either domestic or wild terrestrial animals	Test carried out in humans
The study includes the use of at least one BLCM for evaluation of diagnostic performance	No BLCMs were used in the study

**Table 2:** Characteristics of the studies included in the systematic review.

	Category	Number of articles (25)	Number of trials (71)
Origin of the study	Europe	11	44
	South America	4	13
	North America	2	3
	Africa	4	5
	Asia	4	6

Animal species	Bovine	19	63
	Wild boar	1	2
	Elk/deer	1	2
	Swine	1	1
	Bison	1	1
	Meerkat	1	1
	Badger	1	1

Conditional dependence	Yes	20	49
	No	11	22

Number of populations	1	15	42
	2	5	19
	Multiple	5	10

Number of tests	1	2	6
	2	5	17
	Multiple	18	48

Diagnostic test	Antemortem	20	61
	(i) Skin test	13	49
	(ii) IFN-*γ*	12	44
	(iii) Serology	13	39
	Postmortem	13	30
	(i) Culture	9	17
	(ii) Direct PCR	10	21
	(iii) Pathology	2	10
	(iv) Meat inspection	5	9
	(v) Culture + Meat inspection	1	3

Source of priors	Unspecified	0	0
	Literature	13	26
	Expert knowledge and literature	7	26
	Weakly-informative	5	19

Software	Win BUGS	13	33
	Open BUGS	7	17
	JAGS	5	21

Test dependence	Skin test-IFN-*γ*	7	26
	Skin test-serology	1	1
	Serology-serology	5	6
	PCR-culture	5	7
	PCR-meat inspection	2	3
	PCR-pathology	1	2

**Table 3:** Average posterior median (APM) and 95% average posterior probability intervals (APPI) of the Se and Sp of antemortem diagnostic tests for tuberculosis in the articles reviewed.

Test	*N* ^†^	Se estimates^‡^	APM and 95% APPI Se estimates (%)^§^	Sp estimates^‡^	APM and 95% APPI Sp estimates (%)^§^	Species
**Skin test (PPD-based)**	13	49	66.3 (52.5–74.6)	49	99.1 (98.6–99.5)	
**Cervical**	10	43	66.1 (49.3–72.0)	43	99.2 (98.7–99.5)	Cattle
SIT standard interpretation	6	12	63.9 (46.6–84.0)	12	96.9 (96.3–97.4)	Cattle
SIT severe interpretation	6	10	69.8 (46.0–91.8)	10	99.4 (98.8–99.9)	Cattle
SICCT standard interpretation	5	13	57.5 (52.5–66.6)	13	99.1 (98.2–99.7)	Cattle
SICCT severe interpretation	4	8	70.4 (66.8–75.6)	8	99.2 (98.7–99.6)	Cattle
**Caudal fold**	3	6	72.9 (57.6–86.6)	6	78.7 (64.3–94.1)	Cattle, bison
**IFN- ** *γ * **blood test**	12	44	78.1 (70.5–89.7)	44	89.3 (86.9–91.7)	
BOVIGAM (OD 0.05)	5	10	88.7 (80.1–96.2)	10	88.1 (85.6–91.1)	Cattle
BOVIGAM (OD 0.1)	7	23	75.1 (69.1–81.3)	23	88.2 (86.6–89.6)	Cattle
BOVIGAM (OD 0.2)	1	1	83.3 (74.2–93.5)	1	23.5 (17.4–30.3)	Cattle
IDvet	1	3	49.0 (24.9–94.1)	3	97.9 (97.4–98.4)	Cattle
Antigen TB-feron (OD 0.1)	1	1	97.0 (86.0–100)	1	97.0 (91.4–98.5)	Cattle
ESAT6/CFP10	2	5	66.0 (61.7–72.5)	5	95.3 (95.0–95.5)	Cattle
In-house	1	1	79.9 (68.8–89.5)	1	95.0 (91.4–98.5)	Badger
**Serology test**	13	39	52.2 (39.9–67.4)	39	95.5 (92.0–98.8)	
ELISA	6	15	53.9 (33.1–73.4)	15	95.5 (91.7–98.8)	Cattle, wild boar
Multiplex immunoassay	5	16	51.3 (45.8–56.4)	16	98.7 (97.4–99.5)	Cattle, elk, badger, meerkat, bison
FPA	3	5	38.0 (21.0–54.7)	5	94.0 (92.0–95.0)	Cattle, bison, elk
LST	1	2	71.0 (55.0–84.0)	2	79.0 (76.0–81.0)	Elk
Rapid lateral flow	1	1	93.0 (31.0–99.0)	1	99.0 (95.0–100)	Cattle

^†^Number of articles that evaluate each test. ^‡^Number of trials from which sensitivity and specificity estimates were extracted. ^§^Sensitivity and specificity average posterior median and 95% average posterior probability interval of each test. Nature of the test is indicated in bold.

**Table 4:** Average posterior median (APM) and 95% average posterior probability intervals (APPI) of the Se and Sp of postmortem diagnostic tests for tuberculosis in the articles reviewed.

Test	*N* ^†^	Se estimates^‡^	APM and 95% APPI Se estimates (%)^§^	Sp estimates^‡^	APM and 95% APPI Sp estimates (%)^§^	Species
**Culture**	9	17	79.2 (64.8–88.7)	17	99.8 (98.0–100)	
Tissue	8	15	79.9 (65.0–94.0)	15	99.9 (99.3–100)	Cattle, wild boar, swine, meerkat, badger
Milk and blood	1	2	15.0 (6.6–30.0)	2	97.5 (88.0–100)	Cattle
**Direct PCR**	10	21	80.6 (54.4–92.3)	21	97.6 (95.0–99.7)	
Tissue	9	19	82.9 (69.1–92.3)	19	97.6 (95.4–99.7)	Cattle, wild boar, swine,
Milk and blood	1	2	48.5 (27.0–77.5)	2	93.0 (69.0–99.5)	Cattle
**Meat inspection**	5	9	53.7 (49.9–54.8)	9	99.1 (98.4–99.7)	Cattle
**Histopathology**	2	10	77.8 (55.2–94.6)	10	99.0 (96.8–100)	Cattle
**Culture** **+** **meat inspection**	1	3	74.6 (62.0–87.1)	3	98.5 (97.7–99.2)	Cattle

^†^Number of articles that evaluate each test. ^‡^Number of trials from which sensitivity and specificity estimates were extracted. ^§^Sensitivity and specificity average posterior median and 95% average posterior probability interval of each test. Nature of the test is indicated in bold.

## Data Availability

All data supporting the findings of this study are included in the main article and the supplementary materials.

## Supplementary Materials

Supplementary Materials Supplementary Material 1: Overlap in-house function performed in R to estimate the percentage of overlap between prior and posterior distributions. Supplementary Material 2: Correlation plots between sample size and posterior PIW (probability interval width) for sensitivity and specificity. Supplementary Material 3: Prior and posterior distributions of sensitivities of antemortem and postmortem diagnostic tests for tuberculosis based on informative priors.
