# Supplementary material for: Accuracy of Tests for Diagnosis of Animal Tuberculosis: Moving Away from the Golden Calf (and towards Bayesian Models)
Source: Transbound Emerg Dis. 2023 Feb 21;2023:7615716. doi: 10.1155/2023/7615716 (PMC12017179; doi:10.1155/2023/7615716)
Supplement: Supplementary Materials — Supplementary Material 1: Overlap in-house function performed in R to estimate the percentage of overlap between prior and posterior distributions. Supplementary Material 2: Correlation plots between sample size and posterior PIW (probability interval width) for sensitivity and specificity. Supplementary Material 3: Prior and posterior distributions of sensitivities of antemortem and postmortem diagnostic tests for tuberculosis based on informative priors. [file 7615716.f1.zip › SuppInfo_1.pdf]

Supplementary Material 1

Accuracy of Tests for Diagnosis of Animal Tuberculosis: Moving Away from the Golden Calf (and towards Bayesian Models)

Alberto Gomez-Buendia[1], Pilar Pozo[1], Catalina Picasso-Risso[2,3], Adam Branscum[4], Andres Perez[2], Julio Alvarez[1,5*]

[1] VISAVET Health Surveillance Centre, Universidad Complutense de Madrid, Spain

[2] Veterinary Population Medicine Department, University of Minnesota, St Paul Minnesota, United States

[3] Facultad de Veterinaria, Universidad de la República, Montevideo, Uruguay

[4] Biostatistics Program, Oregon State University, Corvallis Oregon, United States

[5] Departamento de Sanidad Animal, Facultad de Veterinaria, Universidad Complutense de Madrid, Madrid, Spain

[*] **Correspondence:**

Dr. Julio Álvarez

jalvarez@visavet.ucm.es

Overlap in-house function performed in R to estimate the percentage of overlap between prior and posterior distributions

```
#a and b are the parameters of the first beta distribution

f1 = function(x) {

dbeta(x, a, b)

}

#c and d are the parameters of the first beta distribution

f2 = function(x) {

dbeta(x, c, d)

}

#overlap function

overlap = function(f,g, from=0, to=1) {

minf = function(x) return(pmin(f(x), g(x)))

integrate(minf, from, to)}

#check that the overlap of f1 with itself is 1

overlap(f1, f1)

#same for f2

overlap(f2, f2)

o12 = overlap(f1, f2)

#this is the overlap value between the two (prior and posterior) beta distributions:

o12$value
```
