# Supplementary material for: Accuracy of Tests for Diagnosis of Animal Tuberculosis: Moving Away from the Golden Calf (and towards Bayesian Models)
Source: Transbound Emerg Dis. 2023 Feb 21;2023:7615716. doi: 10.1155/2023/7615716 (PMC12017179; doi:10.1155/2023/7615716)

Supplementary Material 2

Accuracy of Tests for Diagnosis of Animal Tuberculosis: Moving Away from the Golden Calf (and towards Bayesian Models)

Alberto Gomez-Buendia[1], Pilar Pozo[1], Catalina Picasso-Risso[2,3], Adam Branscum[4], Andres Perez[2], Julio Alvarez[1,5*]

[1] VISAVET Health Surveillance Centre, Universidad Complutense de Madrid, Spain

[2] Veterinary Population Medicine Department, University of Minnesota, St Paul Minnesota, United States

[3] Facultad de Veterinaria, Universidad de la República, Montevideo, Uruguay

[4] Biostatistics Program, Oregon State University, Corvallis Oregon, United States

[5] Departamento de Sanidad Animal, Facultad de Veterinaria, Universidad Complutense de Madrid, Madrid, Spain

[*] **Correspondence:**

Dr. Julio Álvarez

jalvarez@visavet.ucm.es

Correlation plots between sample size and posterior PIW (probability interval width) for sensitivity and specificity

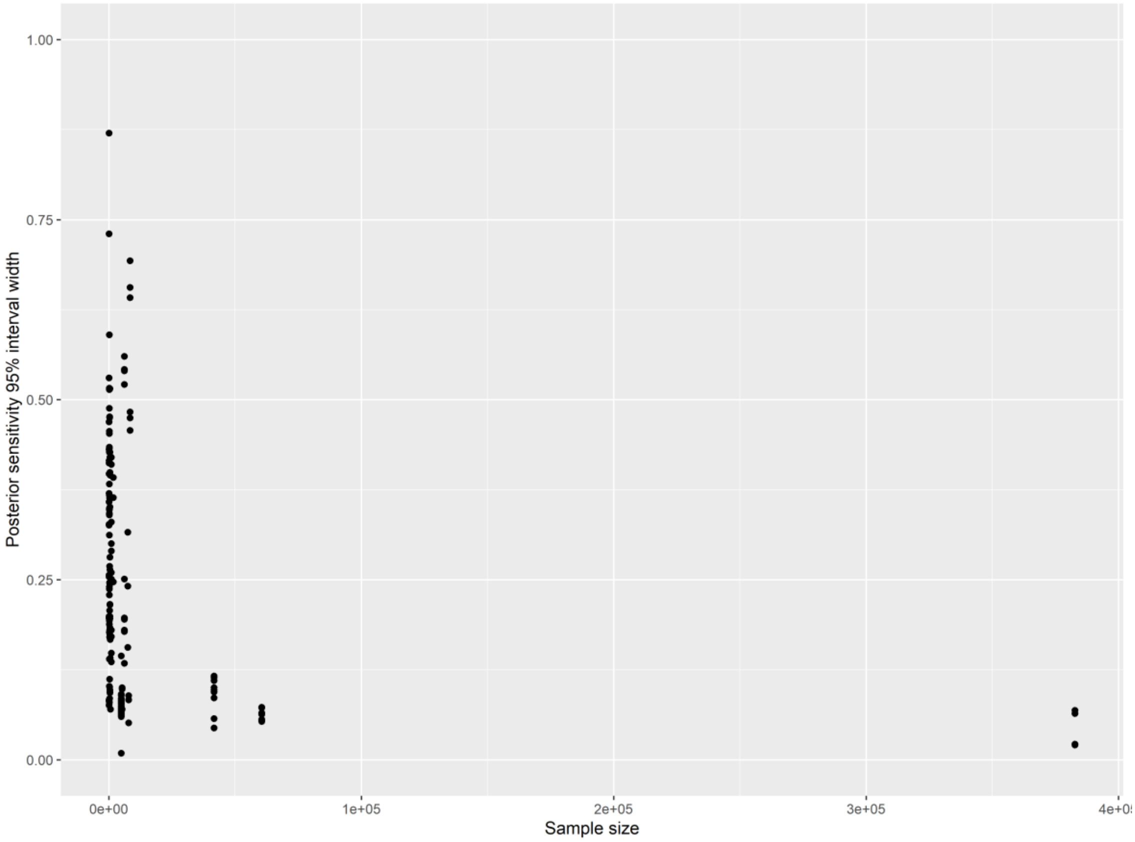

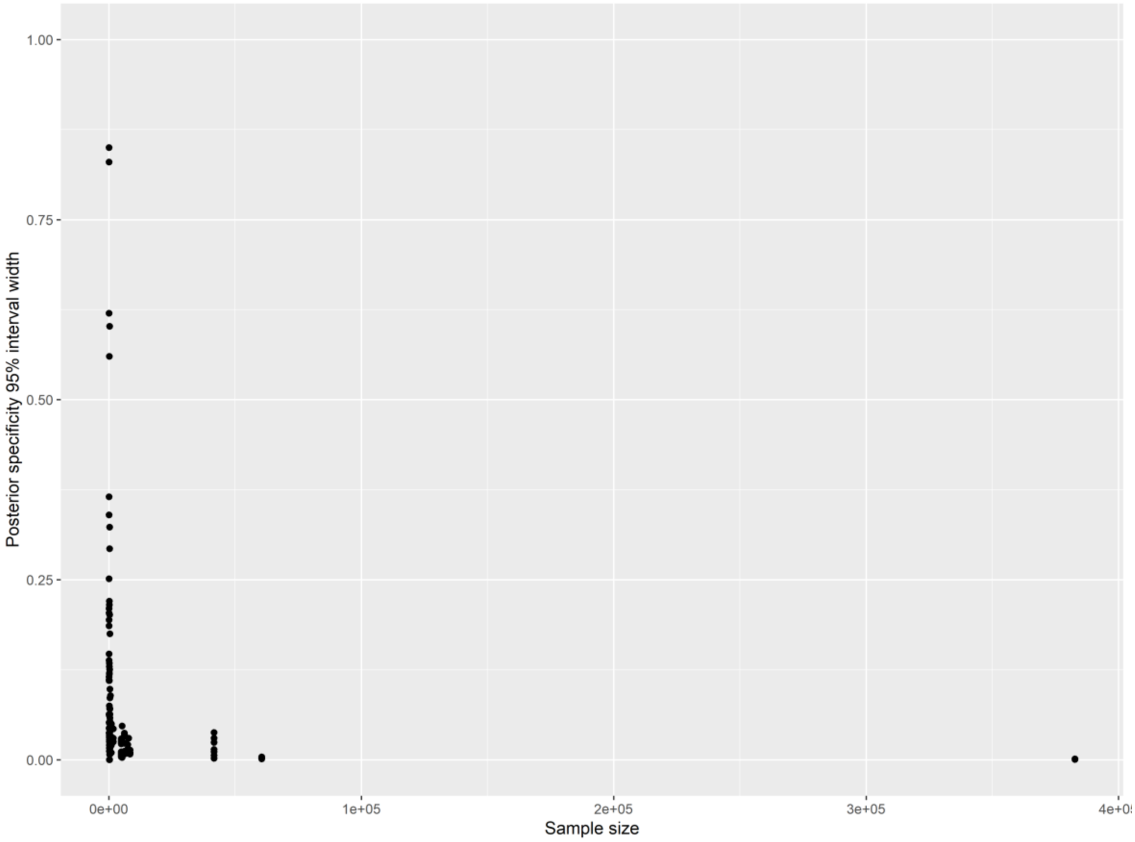

Supplement: Supplementary Materials — Supplementary Material 1: Overlap in-house function performed in R to estimate the percentage of overlap between prior and posterior distributions. Supplementary Material 2: Correlation plots between sample size and posterior PIW (probability interval width) for sensitivity and specificity. Supplementary Material 3: Prior and posterior distributions of sensitivities of antemortem and postmortem diagnostic tests for tuberculosis based on informative priors. [file 7615716.f1.zip › SuppInfo_2.pdf]
