# Supplementary material for: Accuracy of Tests for Diagnosis of Animal Tuberculosis: Moving Away from the Golden Calf (and towards Bayesian Models)
Source: Transbound Emerg Dis. 2023 Feb 21;2023:7615716. doi: 10.1155/2023/7615716 (PMC12017179; doi:10.1155/2023/7615716)
Supplement: Supplementary Materials — Supplementary Material 1: Overlap in-house function performed in R to estimate the percentage of overlap between prior and posterior distributions. Supplementary Material 2: Correlation plots between sample size and posterior PIW (probability interval width) for sensitivity and specificity. Supplementary Material 3: Prior and posterior distributions of sensitivities of antemortem and postmortem diagnostic tests for tuberculosis based on informative priors. [file 7615716.f1.zip › SuppInfo_3.pdf]

Supplementary Material 3

Accuracy of Tests for Diagnosis of Animal Tuberculosis: Moving Away from the Golden Calf (and towards Bayesian Models)

Alberto Gomez-Buendia[1], Pilar Pozo[1], Catalina Picasso-Risso[2,3], Adam Branscum[4], Andres Perez[2], Julio Alvarez[1,5*]

[1] VISAVET Health Surveillance Centre, Universidad Complutense de Madrid, Spain

[2] Veterinary Population Medicine Department, University of Minnesota, St Paul Minnesota, United States

[3] Facultad de Veterinaria, Universidad de la República, Montevideo, Uruguay

[4] Biostatistics Program, Oregon State University, Corvallis Oregon, United States

[5] Departamento de Sanidad Animal, Facultad de Veterinaria, Universidad Complutense de Madrid, Madrid, Spain

[*] **Correspondence:**

Dr. Julio Álvarez

jalvarez@visavet.ucm.es

Prior and posterior distributions of sensitivities of ante-mortem and post-mortem diagnostic tests for tuberculosis based on informative priors

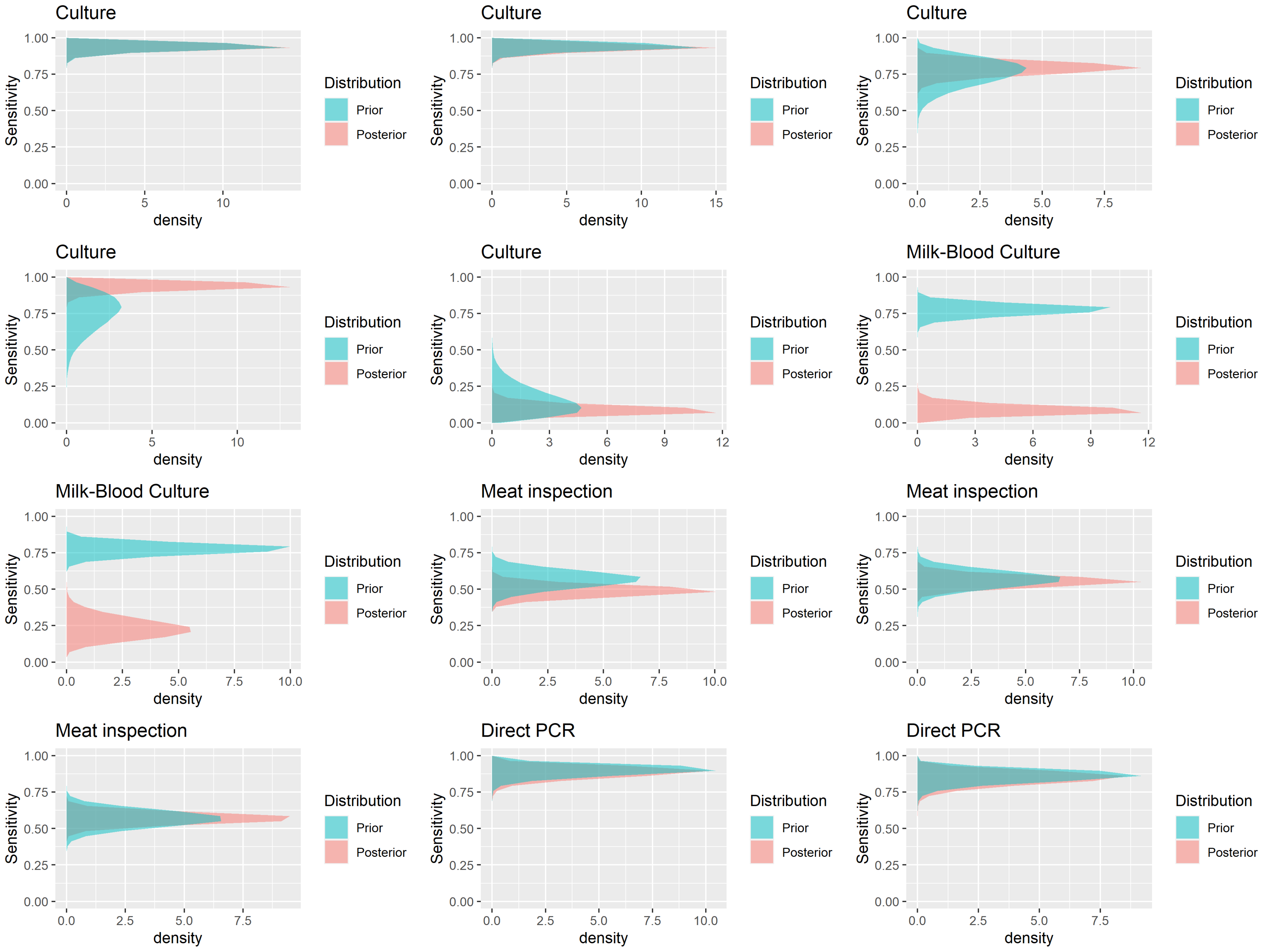
Culture
Culture
Culture
Culture
Culture
Milk-Blood Culture
Milk-Blood Culture
Meat inspection
Meat inspection
Meat inspection
Direct PCR
Direct PCR
Sensitivity
density
Distribution
Prior
Posterior
1.00
0.75
0.50
0.25
0.00
0
5
10
15
0.0
2.5
5.0
7.5
10.0
3
6
9
12

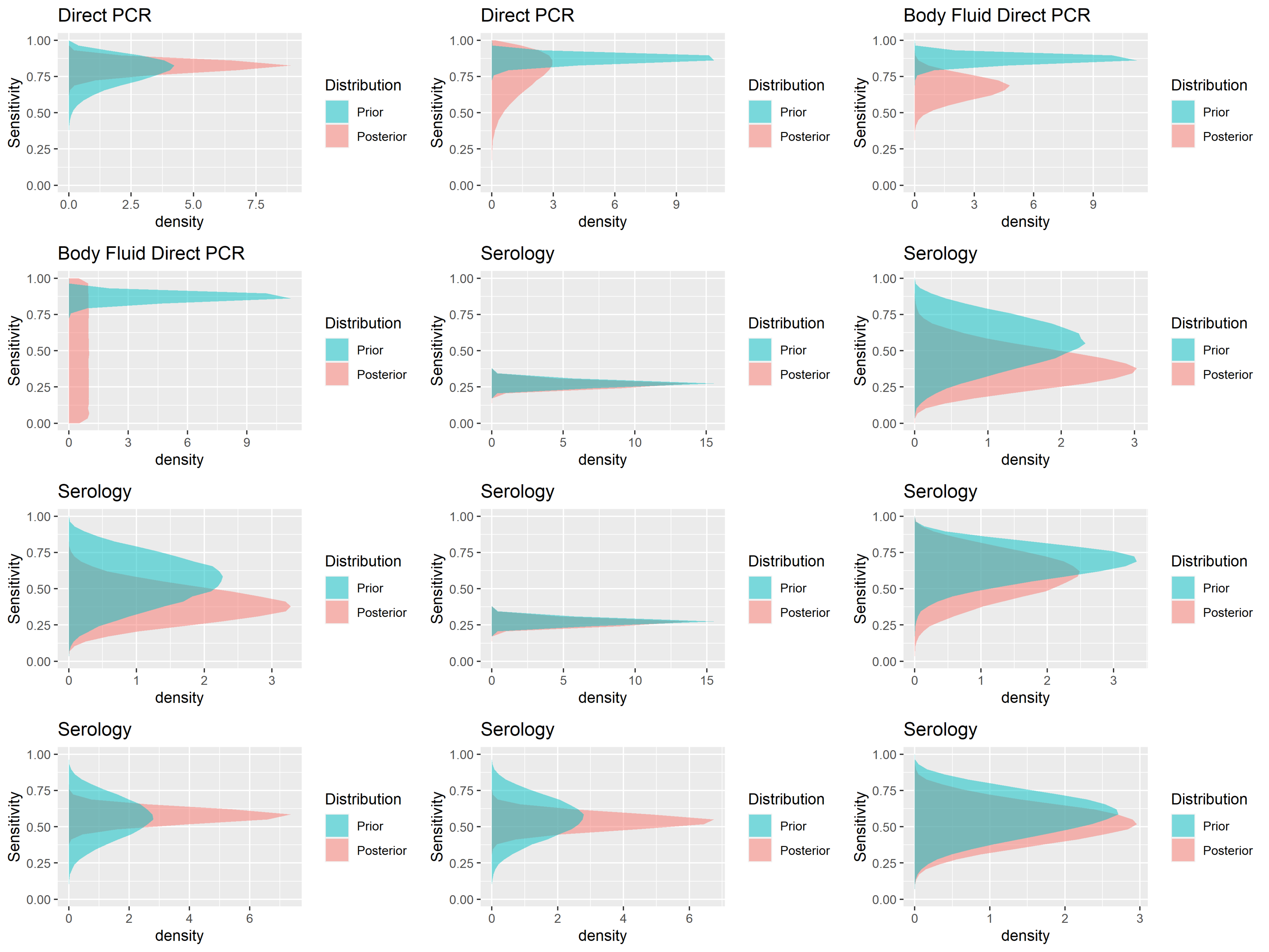

Direct PCR
Direct PCR
Body Fluid Direct PCR
Body Fluid Direct PCR
Serology
Serology
Serology
Serology
Serology
Serology
Serology
Serology
Sensitivity
1.00
0.75
0.50
0.25
0.00
density
0.0
2.5
5.0
7.5
0
3
6
9
0
5
10
15
0
1
2
3
0
2
4
6
Distribution
Prior
Posterior

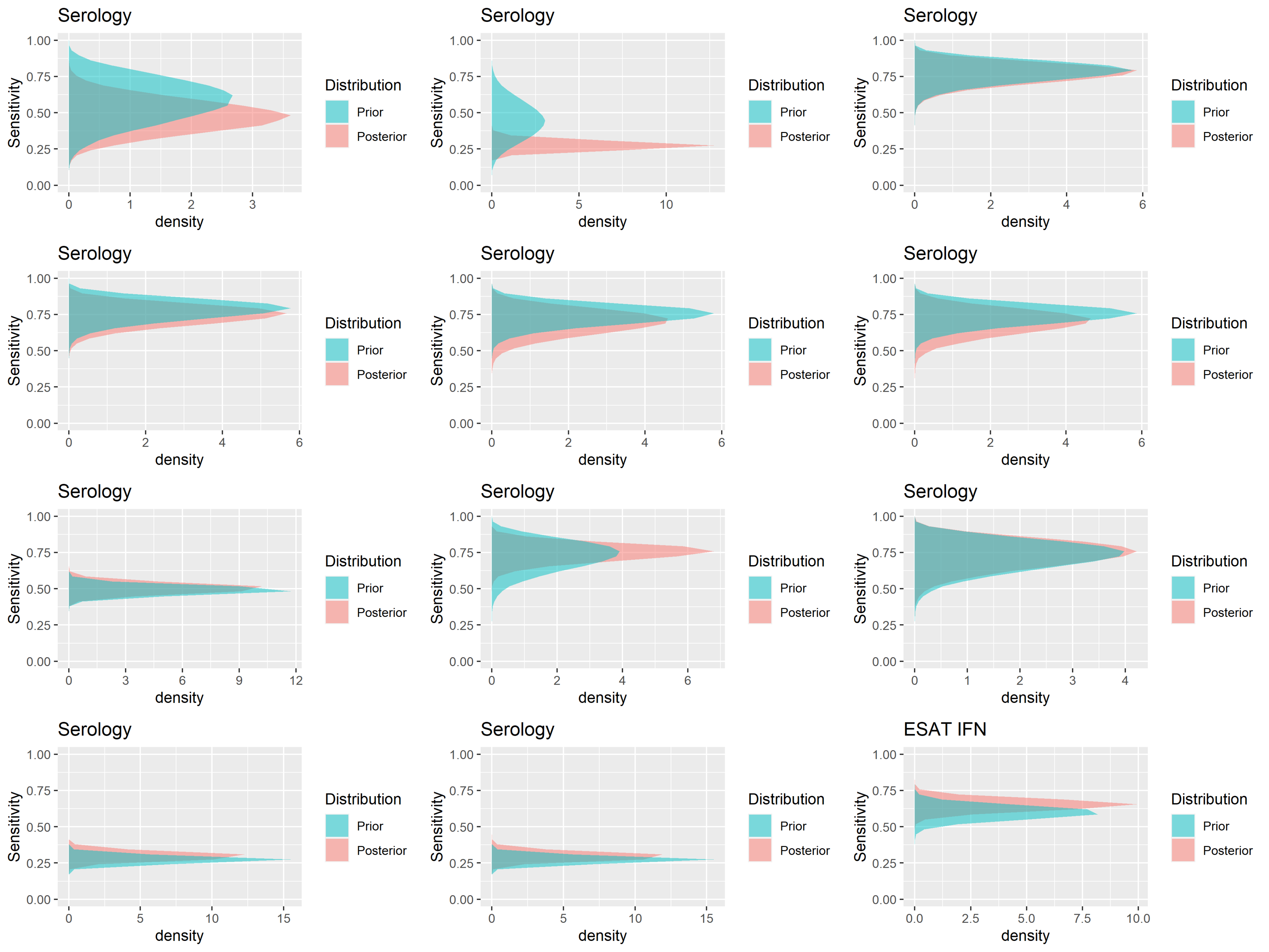

Serology
Serology
Serology
Serology
Serology
Serology
Serology
Serology
Serology
Serology
Serology
ESAT IFN
Sensitivity
density
Distribution
Prior
Posterior
1.00
0.75
0.50
0.25
0.00
0 1 2 3
0 5 10
0 2 4 6
0 3 6 9 12
0 2 4 6
0 1 2 3 4
0 5 10 15
0.0 2.5 5.0 7.5 10.0

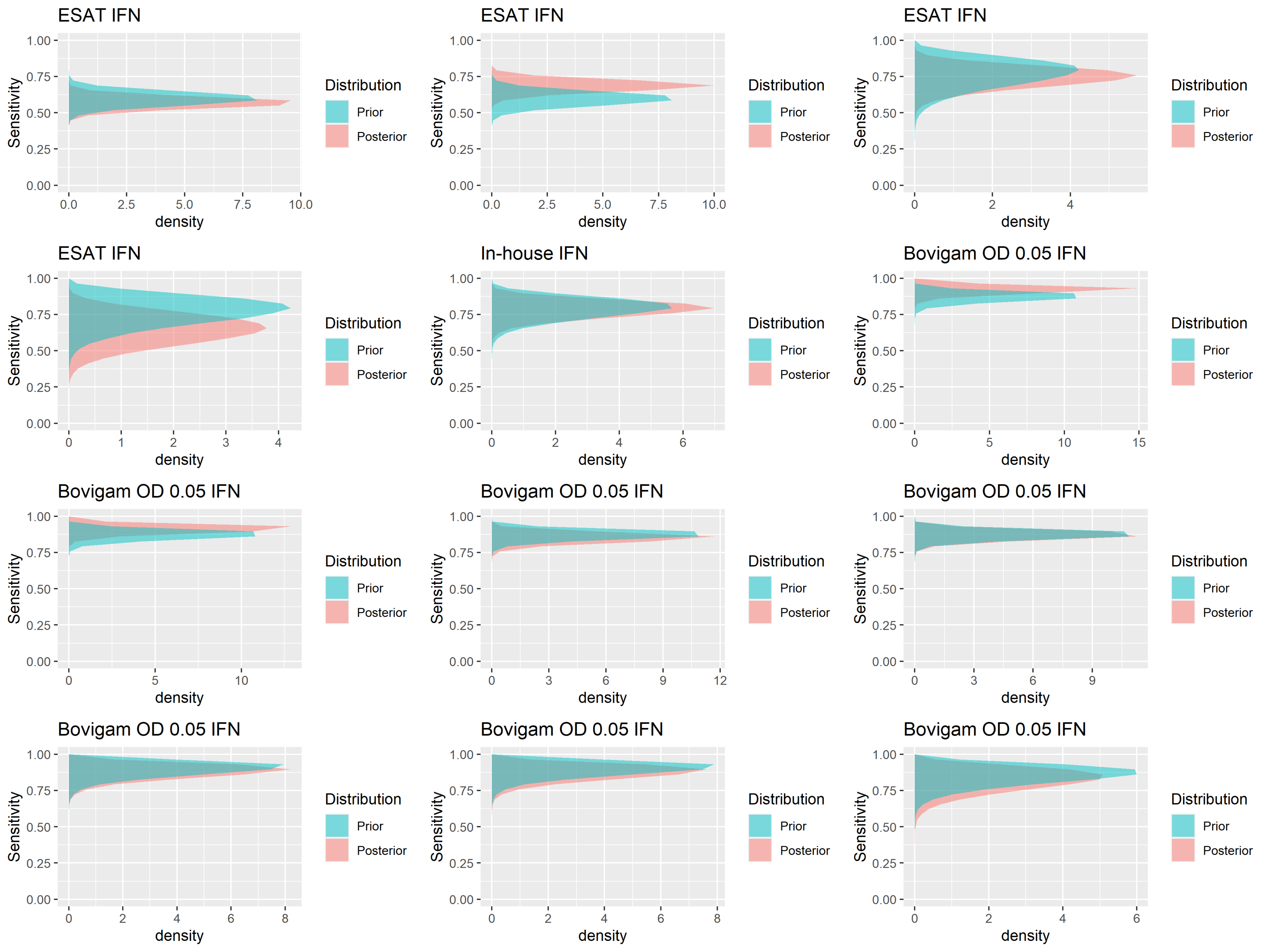
ESAT IFN
ESAT IFN
ESAT IFN
ESAT IFN
In-house IFN
Bovigam OD 0.05 IFN
Bovigam OD 0.05 IFN
Bovigam OD 0.05 IFN
Bovigam OD 0.05 IFN
Bovigam OD 0.05 IFN
Bovigam OD 0.05 IFN
Bovigam OD 0.05 IFN
Sensitivity
density
Distribution
Prior
Posterior

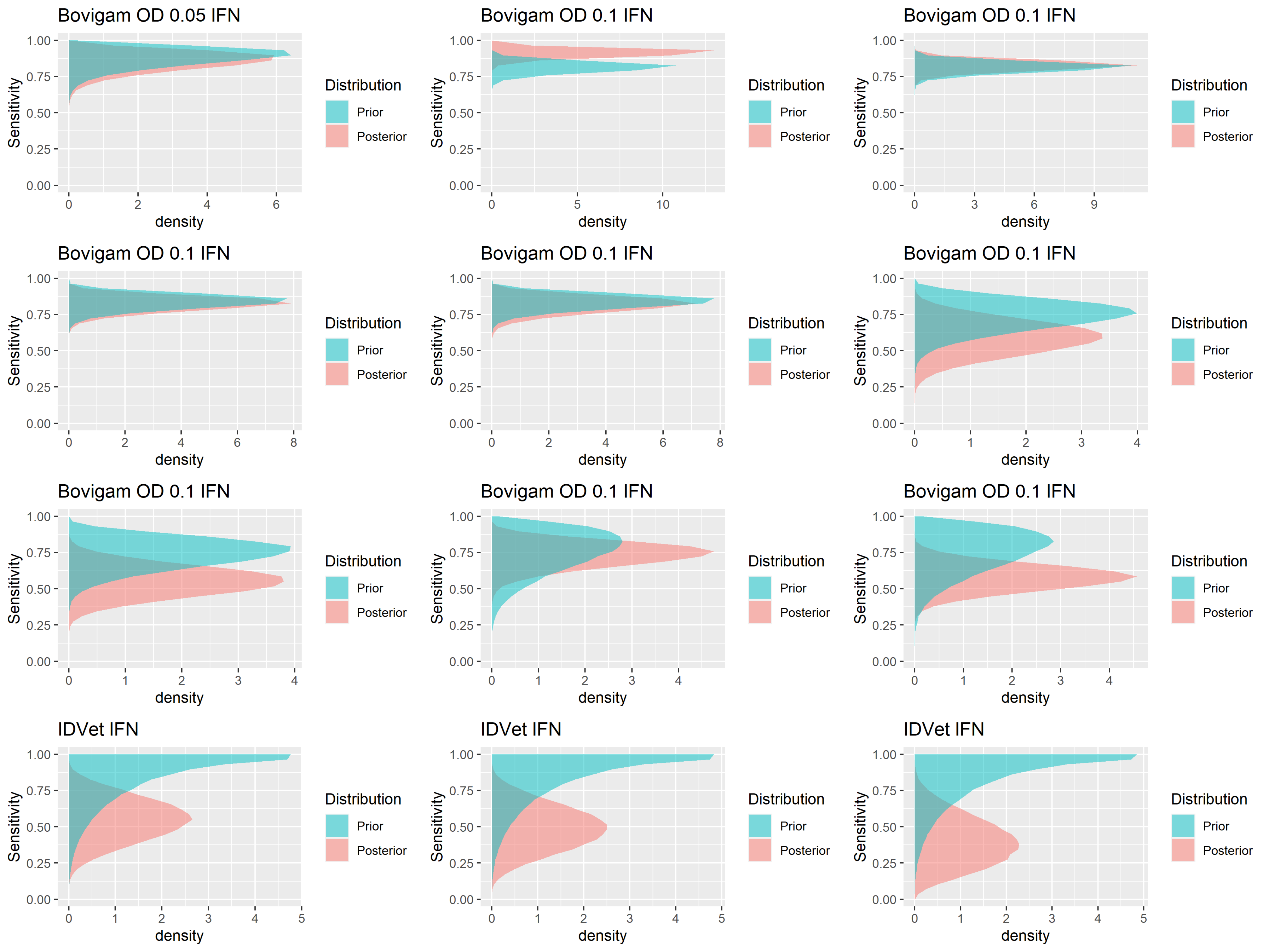

Bovigam OD 0.05 IFN
Bovigam OD 0.1 IFN
Bovigam OD 0.1 IFN
Bovigam OD 0.1 IFN
Bovigam OD 0.1 IFN
Bovigam OD 0.1 IFN
Bovigam OD 0.1 IFN
Bovigam OD 0.1 IFN
Bovigam OD 0.1 IFN
IDVet IFN
IDVet IFN
IDVet IFN
Sensitivity
density
0.00
0.25
0.50
0.75
1.00
Distribution
Prior
Posterior

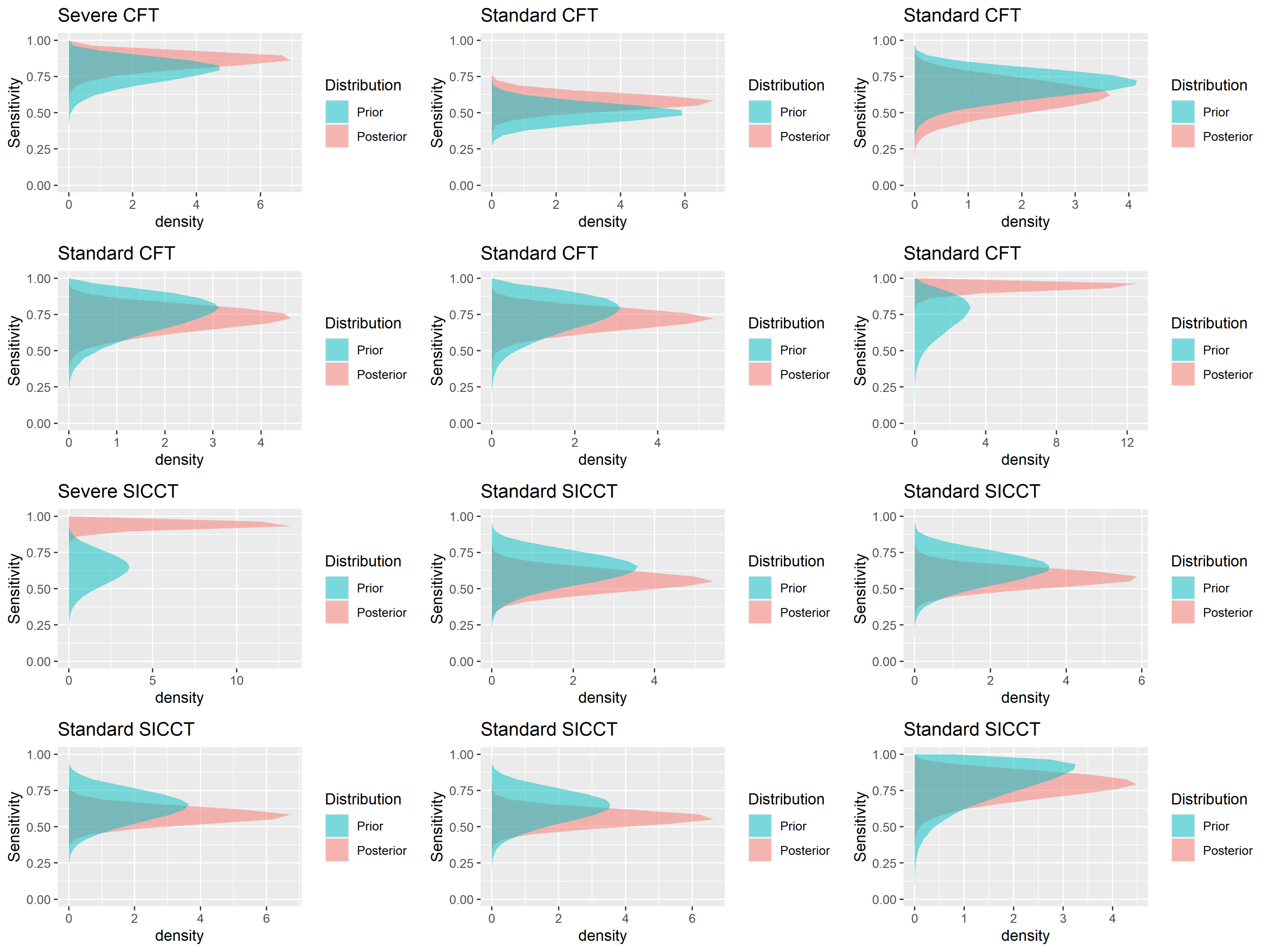
Severe CFT
Standard CFT
Standard CFT
Standard CFT
Standard CFT
Standard CFT
Severe SICCT
Standard SICCT
Standard SICCT
Standard SICCT
Standard SICCT
Standard SICCT
Sensitivity
density
Distribution
Prior
Posterior

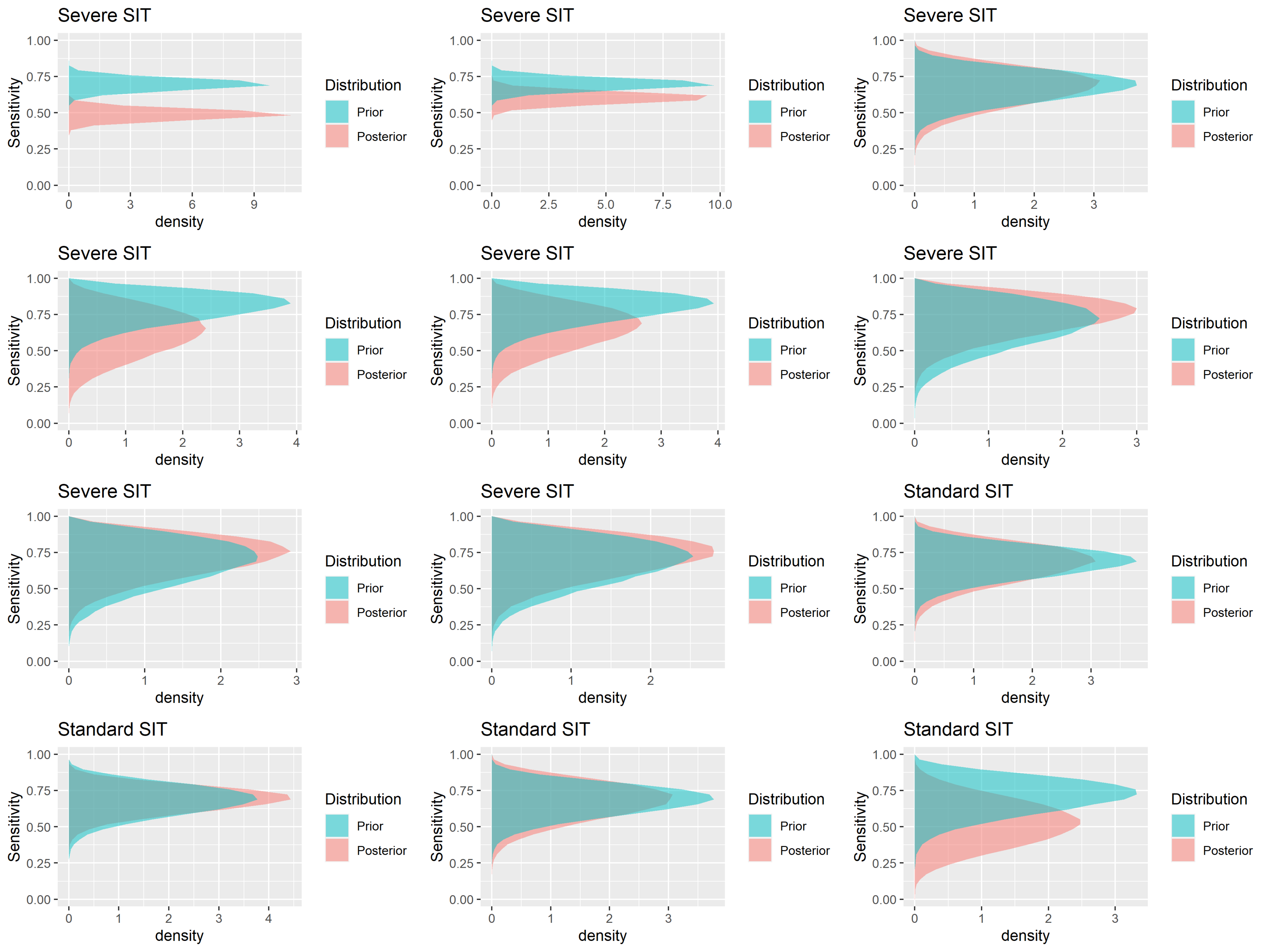

Severe SIT
Sensitivity
1.00
0.75
0.50
0.25
0.00
0
3
6
9
density
Distribution
Prior
Posterior
Severe SIT
Sensitivity
1.00
0.75
0.50
0.25
0.00
0.0
2.5
5.0
7.5
10.0
density
Distribution
Prior
Posterior
Severe SIT
Sensitivity
1.00
0.75
0.50
0.25
0.00
0
1
2
3
density
Distribution
Prior
Posterior
Severe SIT
Sensitivity
1.00
0.75
0.50
0.25
0.00
0
1
2
3
4
density
Distribution
Prior
Posterior
Severe SIT
Sensitivity
1.00
0.75
0.50
0.25
0.00
0
1
2
3
4
density
Distribution
Prior
Posterior
Severe SIT
Sensitivity
1.00
0.75
0.50
0.25
0.00
0
1
2
3
density
Distribution
Prior
Posterior
Severe SIT
Sensitivity
1.00
0.75
0.50
0.25
0.00
0
1
2
3
density
Distribution
Prior
Posterior
Severe SIT
Sensitivity
1.00
0.75
0.50
0.25
0.00
0
1
2
density
Distribution
Prior
Posterior
Standard SIT
Sensitivity
1.00
0.75
0.50
0.25
0.00
0
1
2
3
density
Distribution
Prior
Posterior
Standard SIT
Sensitivity
1.00
0.75
0.50
0.25
0.00
0
1
2
3
4
density
Distribution
Prior
Posterior
Standard SIT
Sensitivity
1.00
0.75
0.50
0.25
0.00
0
1
2
3
density
Distribution
Prior
Posterior
Standard SIT
Sensitivity
1.00
0.75
0.50
0.25
0.00
0
1
2
3
density
Distribution
Prior
Posterior

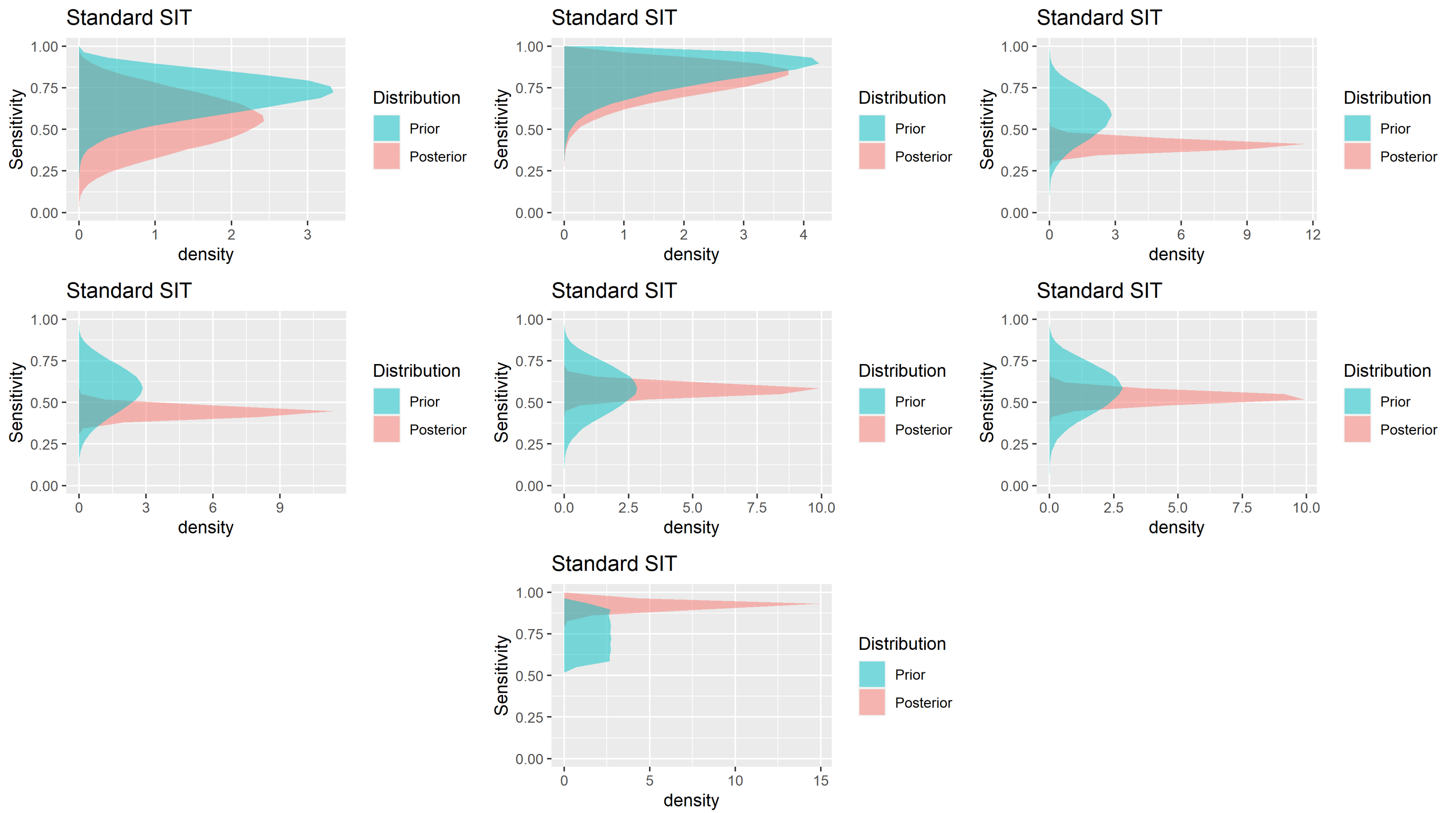
Standard SIT
Sensitivity
1.00
0.75
0.50
0.25
0.00
0
1
2
3
density
Distribution
Prior
Posterior
Standard SIT
Sensitivity
1.00
0.75
0.50
0.25
0.00
0
1
2
3
4
density
Distribution
Prior
Posterior
Standard SIT
Sensitivity
1.00
0.75
0.50
0.25
0.00
0
3
6
9
12
density
Distribution
Prior
Posterior
Standard SIT
Sensitivity
1.00
0.75
0.50
0.25
0.00
0
3
6
9
density
Distribution
Prior
Posterior
Standard SIT
Sensitivity
1.00
0.75
0.50
0.25
0.00
0.0
2.5
5.0
7.5
10.0
density
Distribution
Prior
Posterior
Standard SIT
Sensitivity
1.00
0.75
0.50
0.25
0.00
0.0
2.5
5.0
7.5
10.0
density
Distribution
Prior
Posterior
Standard SIT
Sensitivity
1.00
0.75
0.50
0.25
0.00
0
5
10
15
density
Distribution
Prior
Posterior

Prior and posterior distributions of specificities of ante-mortem and post-mortem diagnostic tests for tuberculosis based on informative priors

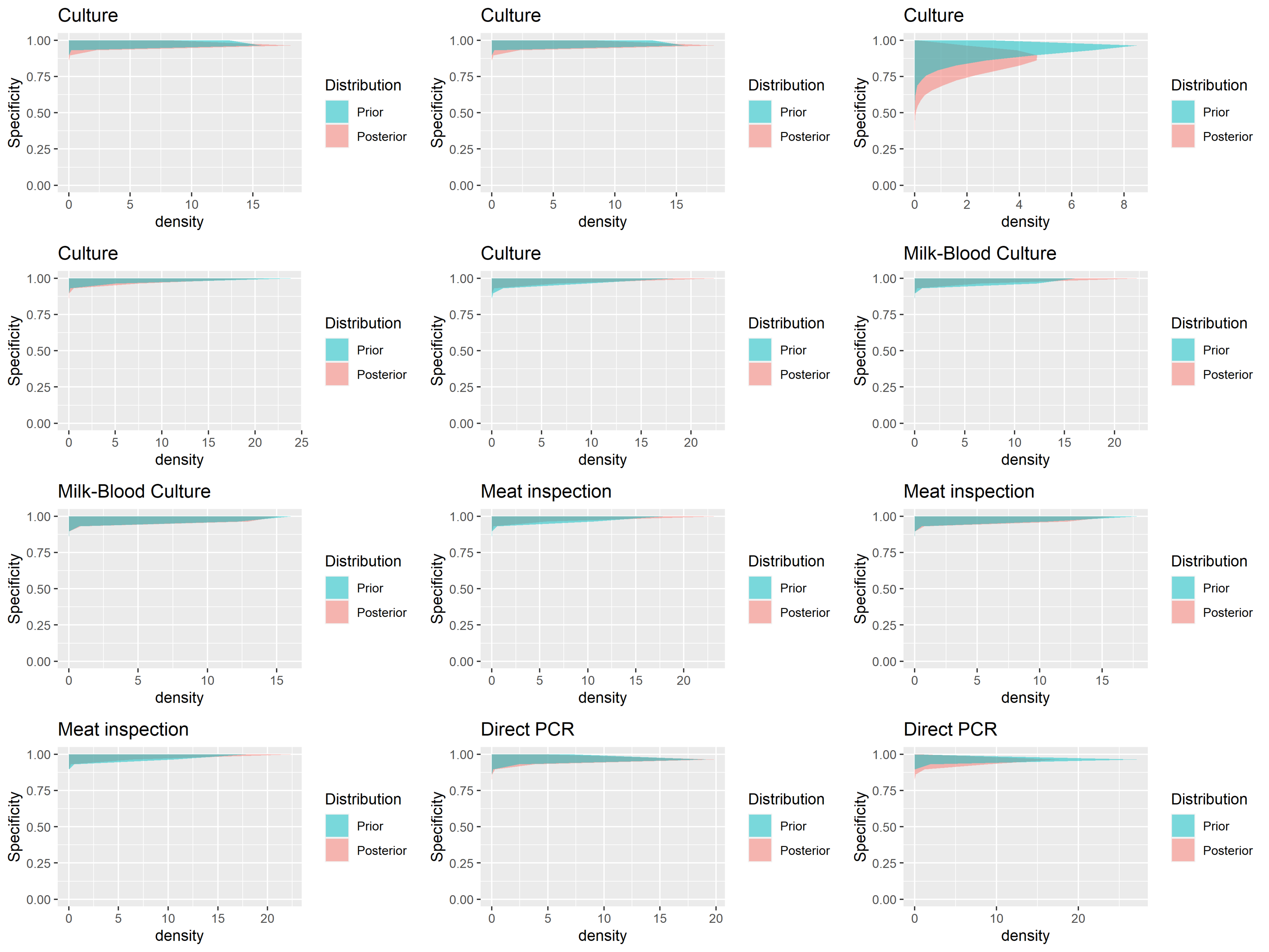
Culture
Specificity
density
Distribution
Prior
Posterior
Culture
Specificity
density
Distribution
Prior
Posterior
Culture
Specificity
density
Distribution
Prior
Posterior
Culture
Specificity
density
Distribution
Prior
Posterior
Culture
Specificity
density
Distribution
Prior
Posterior
Milk-Blood Culture
Specificity
density
Distribution
Prior
Posterior
Milk-Blood Culture
Specificity
density
Distribution
Prior
Posterior
Meat inspection
Specificity
density
Distribution
Prior
Posterior
Meat inspection
Specificity
density
Distribution
Prior
Posterior
Meat inspection
Specificity
density
Distribution
Prior
Posterior
Direct PCR
Specificity
density
Distribution
Prior
Posterior
Direct PCR
Specificity
density
Distribution
Prior
Posterior

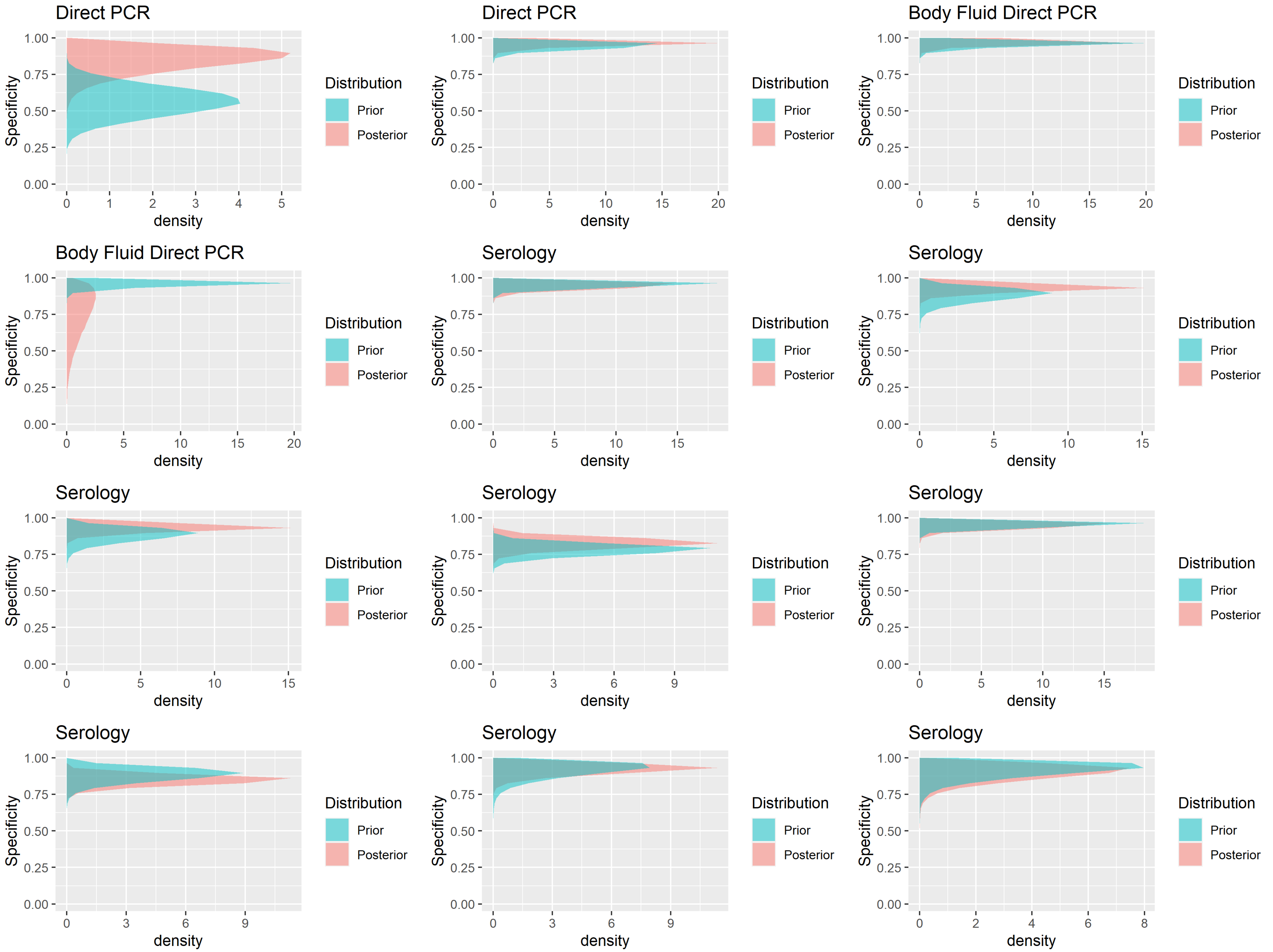
Direct PCR
Direct PCR
Body Fluid Direct PCR
Body Fluid Direct PCR
Serology
Serology
Serology
Serology
Serology
Serology
Serology
Serology
Specificity
density
Distribution
Prior
Posterior

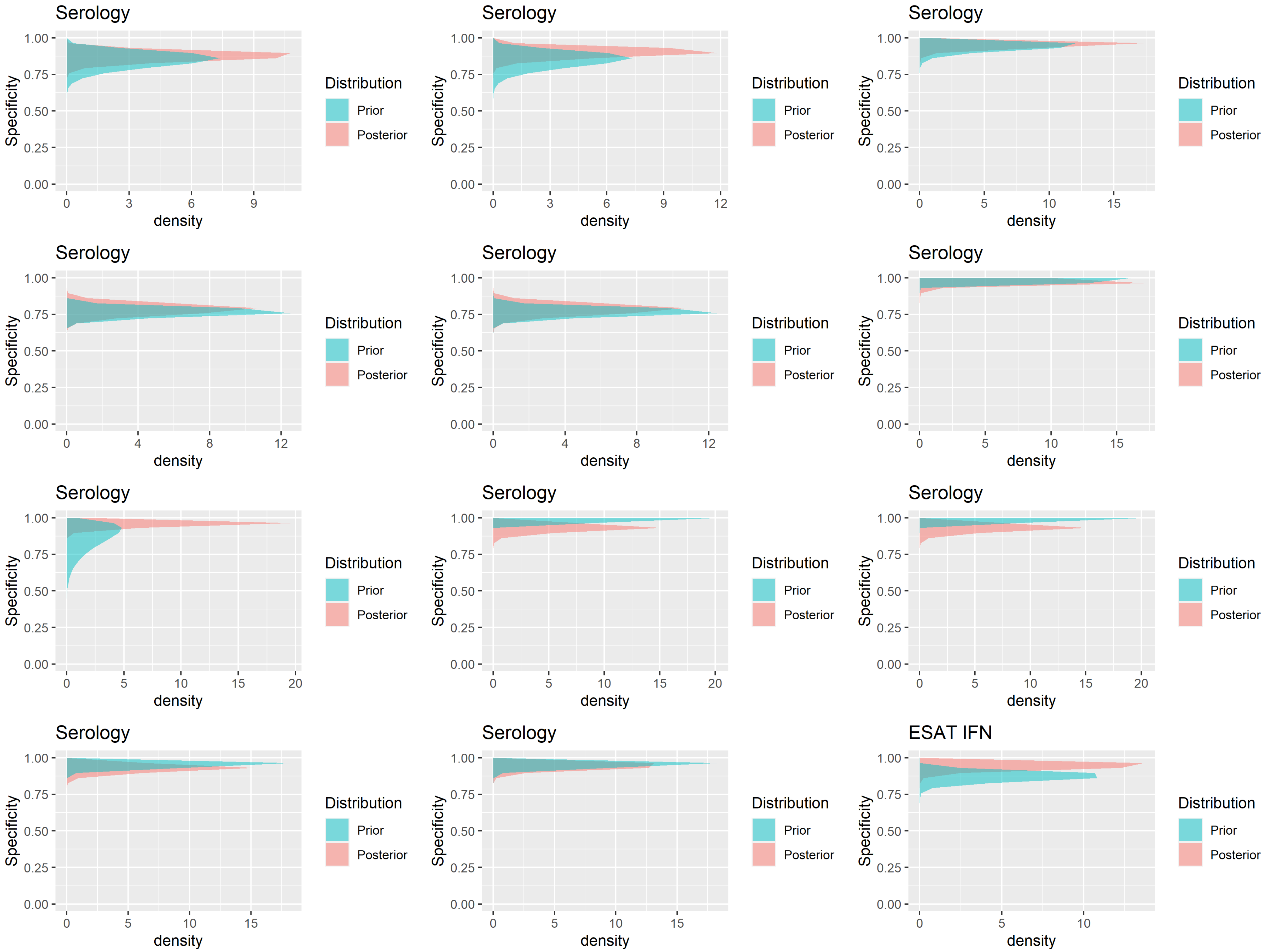
Serology
Specificity
1.00
0.75
0.50
0.25
0.00
density
0
3
6
9
12
Distribution
Prior
Posterior
Serology
0
4
8
12
Serology
0
5
10
15
20
Serology
0
5
10
15
ESAT IFN
0
5
10

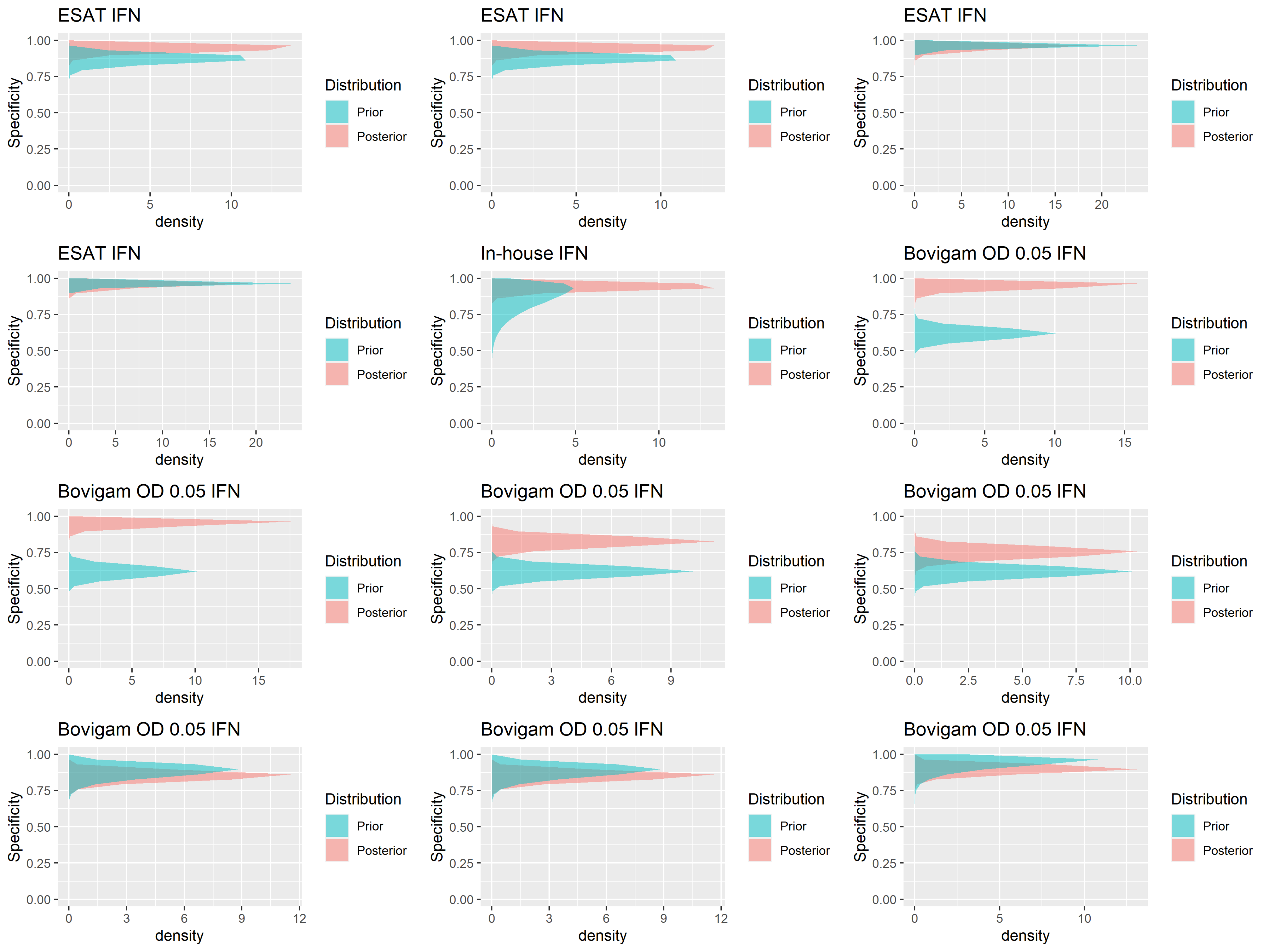
ESAT IFN
ESAT IFN
ESAT IFN
ESAT IFN
In-house IFN
Bovigam OD 0.05 IFN
Bovigam OD 0.05 IFN
Bovigam OD 0.05 IFN
Bovigam OD 0.05 IFN
Bovigam OD 0.05 IFN
Bovigam OD 0.05 IFN
Bovigam OD 0.05 IFN
Specificity
1.00
0.75
0.50
0.25
0.00
density
Distribution
Prior
Posterior

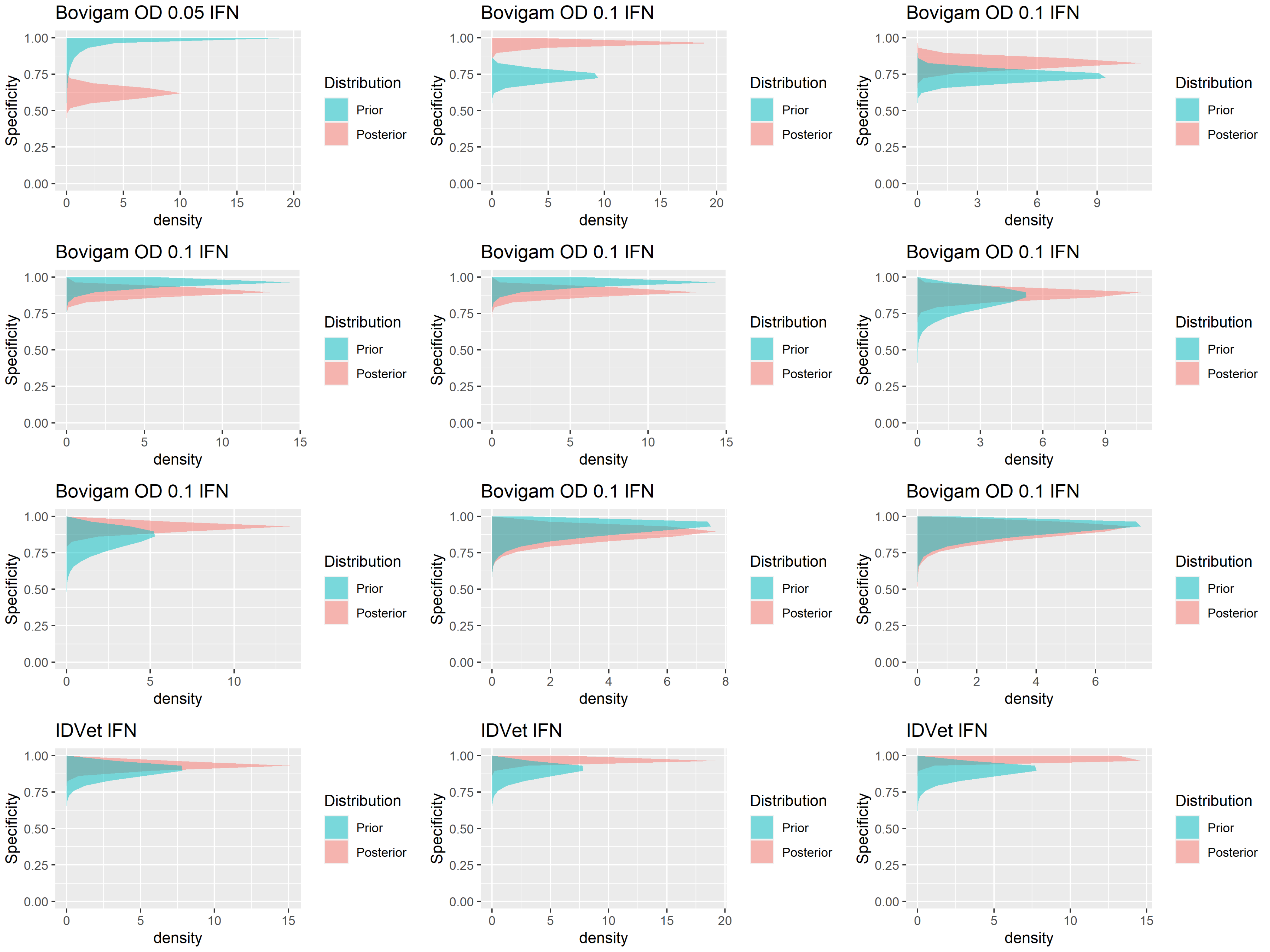

Bovigam OD 0.05 IFN
Bovigam OD 0.1 IFN
IDVet IFN
Specificity
density
Distribution
Prior
Posterior

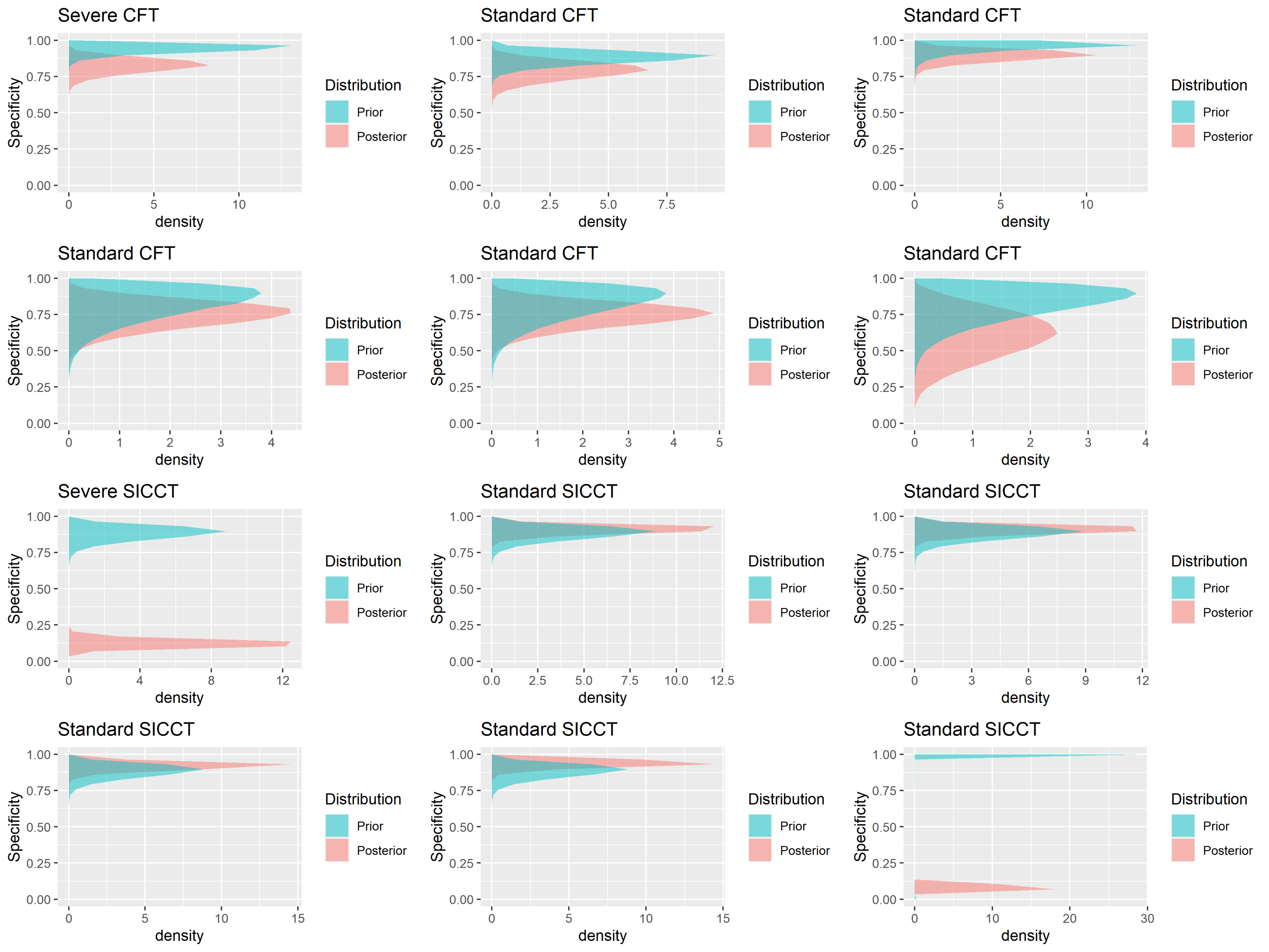
Severe CFT
Standard CFT
Standard CFT
Standard CFT
Standard CFT
Standard CFT
Severe SICCT
Standard SICCT
Standard SICCT
Standard SICCT
Standard SICCT
Standard SICCT
Specificity
density
Distribution
Prior
Posterior
0.00
0.25
0.50
0.75
1.00

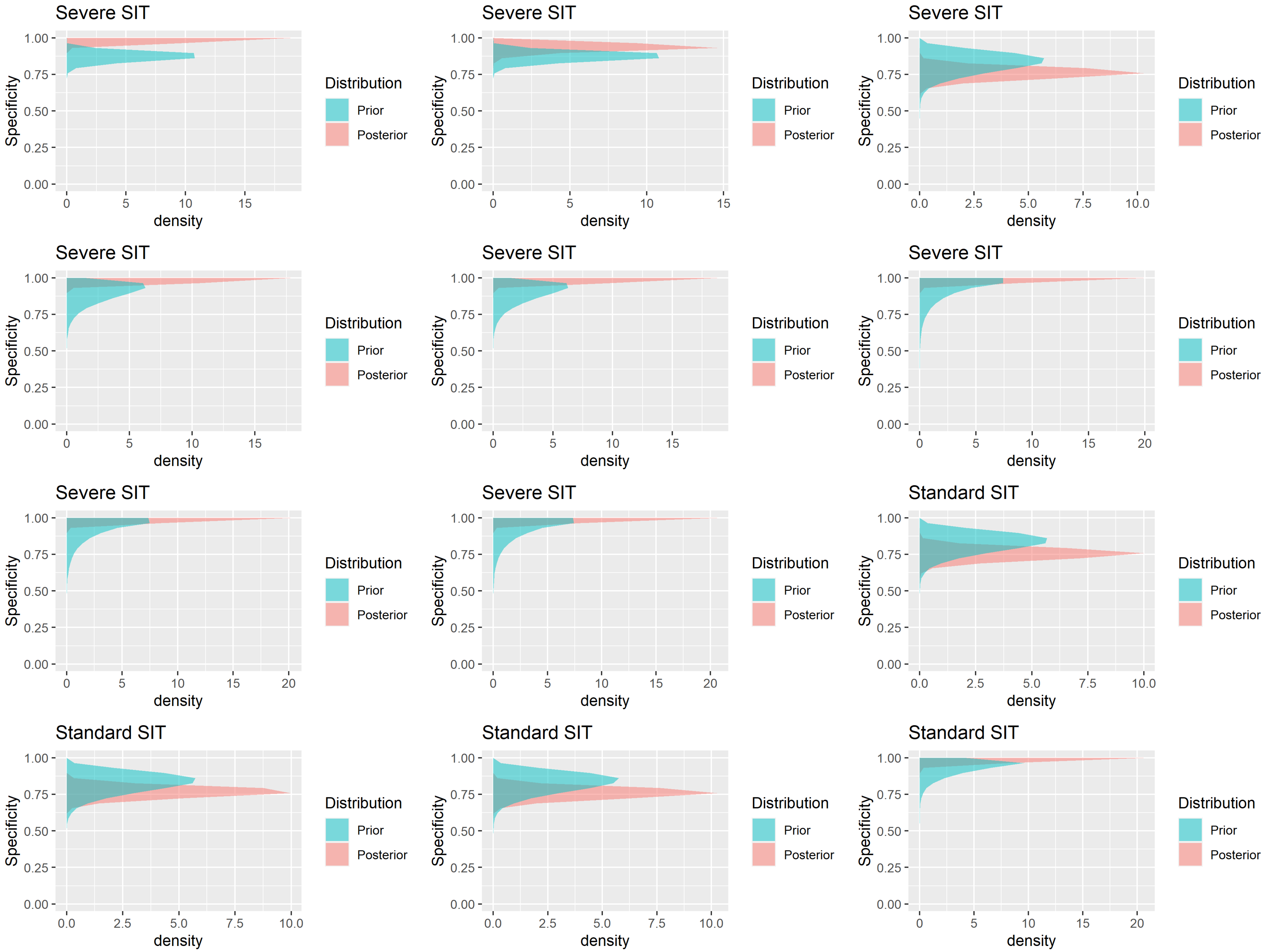
Severe SIT
Specificity
density
Distribution
Prior
Posterior
Severe SIT
Specificity
density
Distribution
Prior
Posterior
Severe SIT
Specificity
density
Distribution
Prior
Posterior
Severe SIT
Specificity
density
Distribution
Prior
Posterior
Severe SIT
Specificity
density
Distribution
Prior
Posterior
Severe SIT
Specificity
density
Distribution
Prior
Posterior
Severe SIT
Specificity
density
Distribution
Prior
Posterior
Severe SIT
Specificity
density
Distribution
Prior
Posterior
Standard SIT
Specificity
density
Distribution
Prior
Posterior
Standard SIT
Specificity
density
Distribution
Prior
Posterior
Standard SIT
Specificity
density
Distribution
Prior
Posterior
Standard SIT
Specificity
density
Distribution
Prior
Posterior

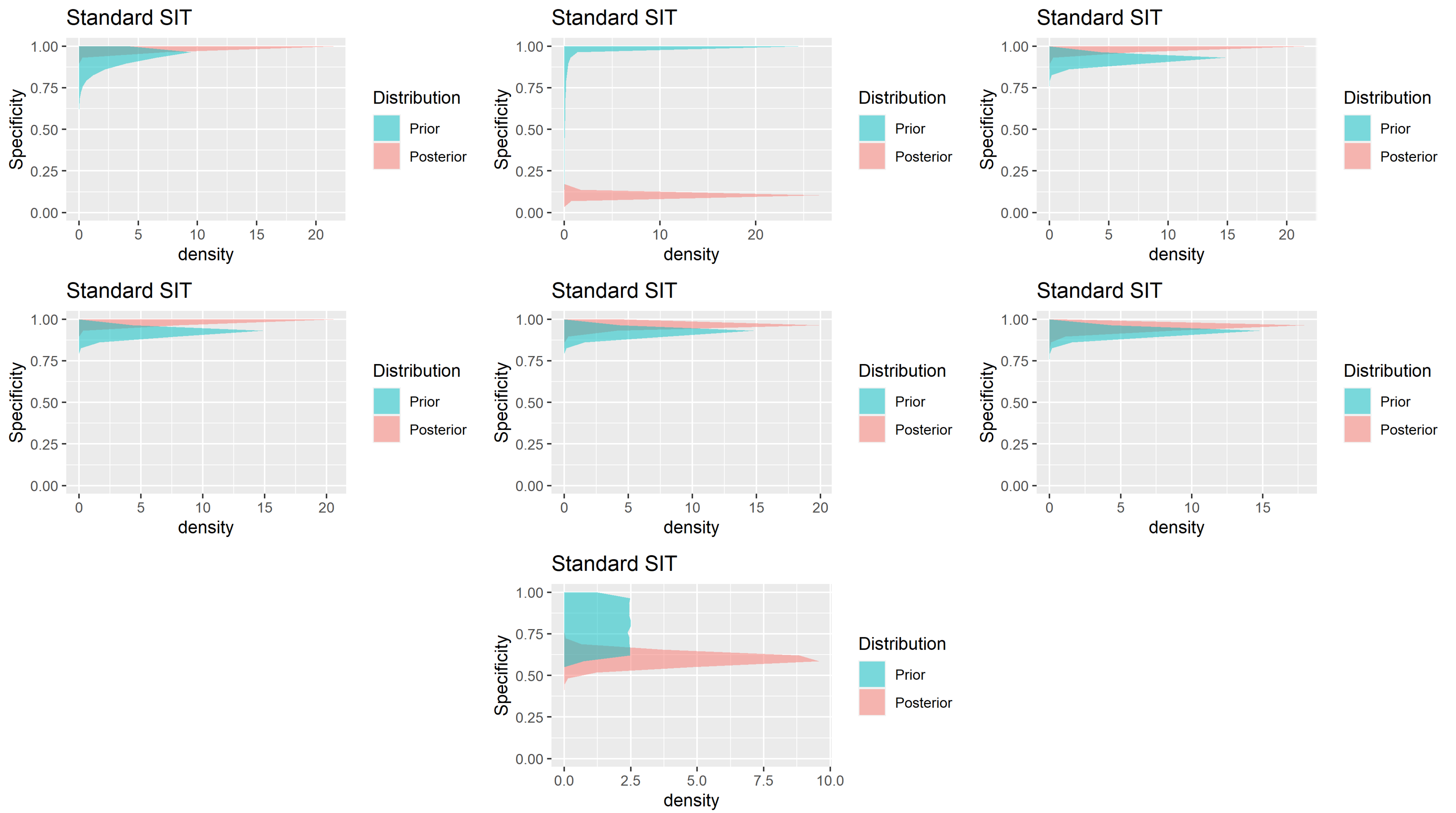

Standard SIT
Specificity
0.00
0.25
0.50
0.75
1.00
density
0
5
10
15
20
Distribution
Prior
Posterior
Standard SIT
Specificity
density
0
10
20
Distribution
Prior
Posterior
Standard SIT
Specificity
density
0
5
10
15
20
Distribution
Prior
Posterior
Standard SIT
Specificity
density
0
5
10
15
20
Distribution
Prior
Posterior
Standard SIT
Specificity
density
0
5
10
15
20
Distribution
Prior
Posterior
Standard SIT
Specificity
density
0
5
10
15
Distribution
Prior
Posterior
Standard SIT
Specificity
density
0.0
2.5
5.0
7.5
10.0
Distribution
Prior
Posterior
